# Advancing Label-Free Imaging Through CARS Microscopy: From Signal Formation to Biological Interpretation

**DOI:** 10.3390/ijms27041990

**Published:** 2026-02-19

**Authors:** Agata Barzowska-Gogola, Emilia Staniszewska-Ślęzak, Joanna Budziaszek, Anna Górska-Ratusznik, Andrzej Baliś, Michał Łucki, Adam Sułek, Barbara Pucelik

**Affiliations:** 1 Łukasiewicz Research Network—Kraków Institute of Technology, 30-418 Kraków, Poland; agata.barzowska-gogola@kit.lukasiewicz.gov.pl (A.B.-G.); emilia.staniszewska-slezak@kit.lukasiewicz.gov.pl (E.S.-Ś.); joanna.budziaszek@kit.lukasiewicz.gov.pl (J.B.); anna.gorska@kit.lukasiewicz.gov.pl (A.G.-R.); andrzej.balis@kit.lukasiewicz.gov.pl (A.B.); michal.lucki90@gmail.com (M.Ł.); 2 Sano Centre for Computational Medicine, 30-054 Kraków, Poland; a.sulek@sanoscience.org

**Keywords:** label-free molecular imaging, Coherent Anti-Stokes Raman Scattering, vibrational biophysics, real-time chemical dynamics, nonlinear optical microscopy

## Abstract

Label-free imaging is becoming ever more important, especially in modern molecular biophysics. This method allows observation of biological structures and dynamics without the alteration caused by dyes or genetic labels. Coherent Anti-Stokes Raman Scattering (CARS) microscopy represents a unique method that utilizes the intrinsic vibrational signatures of biomolecules, thereby transforming the field. Fluorescence-based methods show marked sensitivity, but may cause photobleaching, labeling artifacts, and inadequate biochemical detection. CARS enables chemically specific, real-time imaging of molecular structures, e.g., lipids, proteins and nucleic acids, within their natural environment. Over the past decade, advances in laser technology, detection methods, and computer analysis have turned CARS from a rare optical phenomenon into a useful tool applied in many fields, from basic research on molecular structure to practical biomedical imaging. This review presents the principles of CARS microscopy and the latest achievements in this field, highlighting its impact on molecular and cellular biophysics, as well as exploring the potential of artificial intelligence and multimodal approaches to increase its applications in precision medicine. In this context, CARS serves both a state-of-the-art imaging technique and a means of transforming internal molecular vibrations into information useful in biology and biophysics. In this way, it combines the physical sciences with molecular biology, enabling innovative biomedical research.

## 1. Introduction

### 1.1. The Need for Label-Free Biophysics

Molecular biophysics has entered an era in which the precision of observation is as equally crucial as resolution or sensitivity. The shift from static to dynamic descriptions of multiscale processes has made the multifaced character of numerous established imaging methods a notable limitation rather than a technical inconvenience. The focus has shifted from signal detection to its ability to precisely represent the system’s intrinsic physical behavior [1]. Last decade, the Nobel Prize in chemistry, awarded for advancements in super-resolution fluorescence microscopy, highlights a significant development in the visualization of biological structures beyond traditional limitations [2]. The advancements in cell biology not only significantly altered its understanding but also highlight a critical asymmetry—despite substantial improvements in spatial resolution, the ability to access native chemical dynamics in real time is still limited due to dependence on fluorescent labels. Thus, resolving where molecules are is no longer sufficient without understanding how their intrinsic chemistry evolves in time [3].

Living matter and related biological processes are determined by collective molecular phenomena such as self-organization, phase behavior, and metabolic interactions, which demonstrate significant sensitivity to chemical modifications and environmental stress. In these systems, slight changes from labels can modify energy settings and signaling networks, consequently impairing the mechanisms under investigation [4]. This challenge is particularly evident in molecular biophysics, where interpretation depends on the connection of observable signals to fundamental physical behavior rather than to alternative or bystander markers [4,5,6].

Label-free biophysics addresses this challenge by expanding the role of imaging from molecular labeling toward access to intrinsic molecular properties [7]. Label-free methods utilize endogenous contrast to facilitate the examination of biological systems as physical entities, shaped by their intrinsic chemistry and thermodynamic characteristics [8]. In this emerging framework, super-resolution microscopy and vibrational label-free imaging are not competing paradigms but rather complementary dimensions of insight: one elucidating spatial organization, while the other captures molecular state and dynamics. Nonlinear vibrational techniques, particularly Coherent Anti-Stokes Raman Scattering (CARS), illustrate this convergence by facilitating chemically selective, real-time visualization of endogenous molecular organization without the need for exogenous labels, indicating a conceptual shift in the field towards high-fidelity molecular biophysics [8,9,10].

### 1.2. Limitations of Fluorescence and Classical Raman Spectroscopy

Fluorescence microscopy has become a key tool in molecular biology due to its high sensitivity and ability to identify specific components within cellular contexts [3]. This selectivity depends on the use of exogenous fluorophores or genetically encoded fluorescent tags, which may alter the intrinsic physicochemical properties (e.g., molecular size, charge distribution, steric accessibility) of the labeled molecules. The disturbances are intensified by photophysical constraints associated with fluorescence excitation: (i) photobleaching progressively reduces signal intensity during prolonged observation periods, limiting the feasibility of longitudinal studies and (ii) the phototoxic effects of reactive oxygen species significantly restrict the excitation power and imaging frequency, particularly in live cells and tissues. Fluorescence-based methods inherently face challenges in capturing continuous molecular dynamics over extended durations or under physiologically relevant conditions, particularly in metabolically active or mechanically responsive systems [11,12].

Spontaneous Raman spectroscopy (SRS) offers a distinctive approach by providing label-free chemical specificity through intrinsic vibrational fingerprints [13,14]. This enables direct access to molecular composition and structure while preserving the integrity of chemicals. The low Raman scattering cross-section results in weak signals, necessitating prolonged acquisition times and elevated excitation intensities. The limitations hinder the achievement of adequate temporal resolution and signal-to-noise ratios, rendering classical Raman microscopy unsuitable for observing rapid or transient biological processes in living systems [9,15,16].

The identified limitations collectively indicate a fundamental methodological constraint in molecular imaging. Fluorescence microscopy exhibits high sensitivity; however, it may disrupt the system. Spontaneous Raman spectroscopy maintains chemical authenticity; however, it lacks the temporal resolution required for dynamic studies. In molecular biophysics, the necessity of elucidating both molecular identity and real-time dynamics presents a significant challenge, rather than a justifiable compromise.

### 1.3. Why Label-Free, Real-Time Imaging Is Transformative for Biophysics and Medicine

The increasing emphasis on the dynamic of multiscale biological processes has fundamentally altered the experimental requirements of molecular biophysics. Rather than static descriptions of molecular composition, contemporary questions demand access to time-resolved molecular states that evolve under near-physiological conditions. Label-free, real-time imaging meets this requirement by enabling continuous observation of biochemical and structural dynamics without introducing external perturbations that confound physical interpretation [7,17]. This capability is particularly consequential for systems regulated by membrane organization, metabolic state, etc. In such systems, functional states are defined not by single molecular events but by transient spatial correlations, concentration fluctuations, and dynamic reorganization. Techniques that preserve endogenous molecular contrast while providing sufficient temporal resolution allow these phenomena to be interrogated as physical processes, rather than inferred indirectly from static or labeled observations [18]. From a biomedical point of view, the same principles are applicable. Disease-associated transitions, occurring, i.e., in cancer, often present as gradual alterations in molecular composition or metabolic state occurring prior to noticeable morphological changes [19,20]. Label-free, real-time imaging provides a method for the direct detection and quantification of these transitions, thereby establishing a mechanistic connection between molecular dynamics and functional phenotype [21]. Thus, label-free biophysics offers an interconnected framework that integrates basic physical principles with translational relevance.

### 1.4. CARS as a Game-Changing Technology in Molecular Biophysics

**Figure 1:**
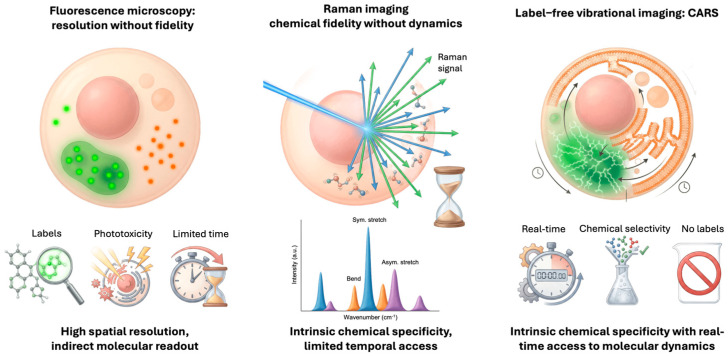
From labeled observations to label-free molecular dynamics: Fluorescence microscopy provides high spatial resolution but relies on exogenous labels that can perturb native molecular behavior (**left**); SRS offers intrinsic chemical specificity but is limited by low signal levels and restricted temporal resolution (**middle**); label-free nonlinear vibrational imaging, exemplified by CARS, bridges this gap by enabling chemically selective, real-time visualization of endogenous molecular organization and dynamics in living systems (**right**).

In the broader context of label-free imaging, CARS is unique in that it balances the need for chemical specificity with the need for living biological samples to respond rapidly. CARS overcomes the inherent sensitivity limitations of SRS by employing coherent nonlinear stimulation of molecular vibrations. This allows one to quickly map specified vibrational modes in situ with spatial resolution. This functionality is valuable for more than simply technical performance [22]. CARS provides direct access to classes of biomolecules, particularly lipids, that are critical for cell architecture, energy balance, and signaling but are underserved by conventional labeling approaches. CARS enables researchers to explore lipid-mediated processes as integral components of cellular physics rather than as supplementary aspects. This is conceivable because CARS can detect their spatial distribution and temporal change without using external probes [23]. In summary, CARS represents a shift in molecular biophysics from proxy-based imaging to a direct observation of endogenous molecular architecture and dynamics, see Figure 1. In this way, it demonstrates how nonlinear vibrational imaging can push the boundaries of the discipline in both theory and application.

## 2. Principles and Scope of CARS

### 2.1. Physical Principles of Coherent Raman Scattering

The first experimental studies on this phenomenon were carried out by Terhune and Marker in 1965 at the Ford Motor Company, an automobile manufacturer [24]. The term CARS was first introduced by Begley, who applied this technique to study nonlinear properties in solid, liquids, and gases [25]. With the advent of high-peak-power pulsed dye lasers, Duncan et al. realized the first CARS microscope in 1982. Their system employed two picosecond lasers in a non-collinear beam configuration, enabling signal detection in the phase-matching direction and the acquisition of two-dimensional images [26]. The next important step in the development of CARS was presented by Zumbush et al. three-dimensional CARS imaging by using tight focusing conditions for spatially and temporally overlapping pump and Stokes laser beam [27]. Developments in the field of optoelectronics have significantly contributed to the development of CARS microscopy as a tool for rapid spectral imaging, thereby enabling the visualization of rapid cellular processes.

CARS is based on the controlled excitation of molecular vibrations by multiple synchronized optical fields. Understanding how this coherence is created is essential to appreciating both the advantages and limitations of this technique. In this section, we present the basic excitation mechanism that leads from the interaction of laser beams to the creation of macroscopic vibration polarization responsible for anti-Stokes emission. Unlike spontaneous Raman scattering—which is weak and incoherent—CARS generates a coherent anti-Stokes signal that is orders of magnitude stronger and directionally emitted [28]. In the CARS process, two laser fields, pump ($\omega_{p}$) and Stokes ($\omega_{S}$), excite a vibrational coherence between the ground and an excited vibrational level of a molecule when the frequency difference satisfies

$$\omega_{p}-\omega_{S}=\omega_{v},$$

where $\omega_{v}$ is the molecular vibrational frequency.

A third field, known as the probe (often identical to the pump), interacts with this vibrational coherence, generating a signal at the anti-Stokes frequency

$$\omega_{aS}=\omega_{pr}+\left(\omega_{p}-\omega_{S}\right).$$

**Figure 2:**
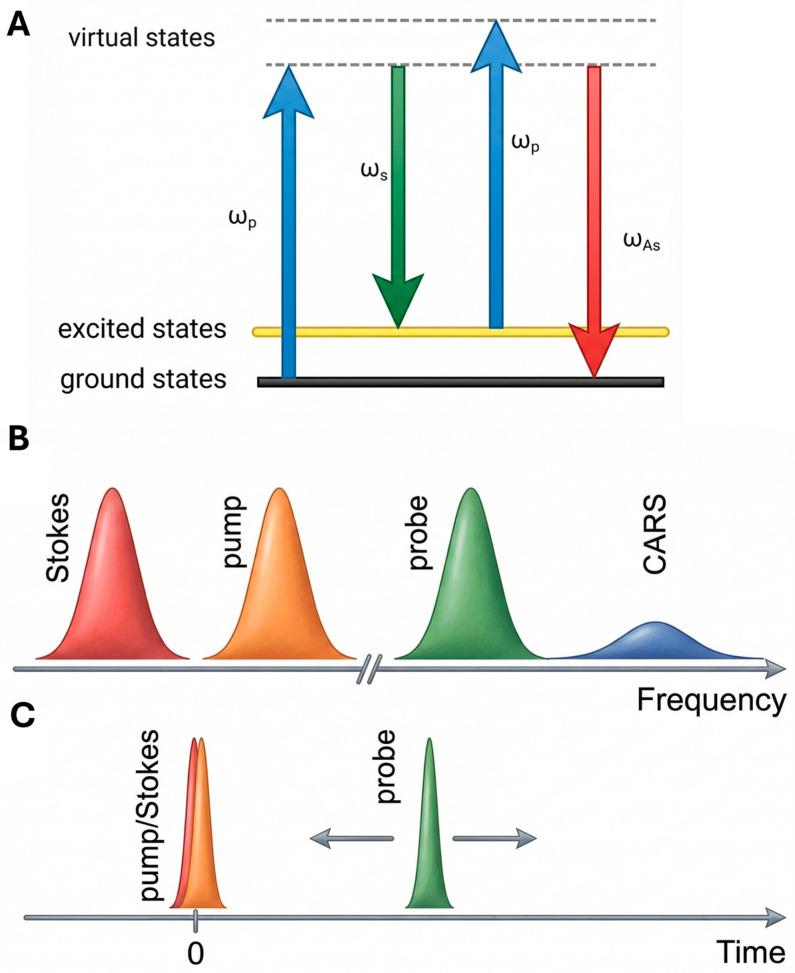
Physical principles and diagrams of CARS excitation: (**A**) Energy level diagram illustrating the CARS process. Two pumping photons (ω_p_) and one Stokes photon (ω_s_) coherently excite molecular vibrations through virtual states, generating vibration coherence, which is then probed by a pumping (or probing) photon, resulting in the emission of an anti-Stokes photon (ω_AS_). (**B**) Frequency-domain representation of the pump, Stokes, and probe fields and the resulting CARS signal shifted toward the blue, emphasizing the spectral separation from the fluorescent background. (**C**) Time-domain excitation scheme showing the temporal overlap of the pump and Stokes pulses at zero delay to generate vibrational coherence, followed by a controlled time delay of the probe pulse, which is the basis for implementing CARS with time resolution and background suppression.

The energy diagram of CARS is shown in Figure 2. Two photons (pump and Stokes) excite a specific vibrational resonance coherently. A third photon (probe) subsequently measures the density of the vibrational resonance. The number of emitted anti-Stokes photons that are energy shifted by that vibrational mode is proportional to the square of the density of the vibrational oscillators, thus yielding the molecular concentration of the target [29].

This photon has higher energy than the pump photon and thus appears at the *anti-Stokes* side of the spectrum [25]. In quantum mechanical terms, the process can be interpreted as a four-wave mixing event involving virtual transitions and coherent polarization of the medium. The macroscopic polarization driving the anti-Stokes field arises from the third-order nonlinear response of the medium and is expressed as

$$P^{\left(3\right)}(t)=\varepsilon_{0}∭\chi^{\left(3\right)}(t-t_{1},t-t_{2},t-t_{3})E(t_{1})E(t_{2})E(t_{3})\;dt_{1}dt_{2}dt_{3}.$$

In the frequency domain, the polarization associated with the anti-Stokes frequency is

$$P^{\left(3\right)}(\omega_{aS})=\varepsilon_{0}\chi^{\left(3\right)}(\omega_{aS};\omega_{p},-\omega_{S},\omega_{pr})E_{p}E_{S}^{*}E_{pr}.$$

This coherent preparation of the vibrational ensemble provides the physical origin of the anti-Stokes signal. However, the way this response is detected differs fundamentally from spontaneous Raman spectroscopy and introduces additional complexity into spectral interpretation. To understand how the measured intensity relates to the intrinsic molecular vibrations, the operational measurement framework must be considered.

### 2.2. Operational Principle and Measurement Model of CARS

While the excitation scheme determines how vibrational coherence is established, interpretation of CARS data ultimately depends on what observable reaches the detector. Unlike spontaneous Raman scattering, where the signal is directly linked to the imaginary component of the susceptibility, the interaction of multiple optical fields with the sample induces a third-order nonlinear polarization (based on four-wave mixing model), which acts as the source of the coherent Raman signal. While vibrational coherence defines how the signal is generated, interpretation of CARS requires understanding what is actually detected. The measured anti-Stokes intensity originates from the third-order nonlinear susceptibility of the medium $\chi^{\left(3\right)}$, which contains both resonant contributions associated with molecular vibrations and non-resonant electronic contributions [30].

In the case of CARS, the experimentally measured signal intensity is proportional to the squared modulus of the total third-order susceptibility, $|\chi^{\left(3\right)}{|}^{2}$ and follows

$$I_{CARS}\propto {|P^{\left(3\right)}(\omega_{aS})|}^{2}\propto {|\chi_{CARS}^{\left(3\right)}|}^{2}I_{p}^{2}I_{S}.$$

where $I_{CARS}$ denotes the detected anti-Stokes intensity and $P^{\left(3\right)}\left(\omega_{aS}\right)$ is the third-order nonlinear polarization oscillating at the anti-Stokes frequency $\omega_{aS}$, which acts as the source of the emitted radiation. $\chi_{CARS}^{\left(3\right)}$ represents the effective third-order nonlinear susceptibility of the medium. The quantities $I_{p}$ and $I_{S}$ correspond to the intensities of the pump and Stokes excitation fields, respectively.

Importantly, the susceptibility of entering this expression is not a purely vibrational quantity. Instead, it comprises both a resonant contribution associated with molecular transitions and a non-resonant term originating from the instantaneous electronic response of the medium. Because these components combine at the level of the nonlinear polarization prior to intensity detection, their mutual interference becomes an inherent property of the measured spectrum. Consequently, the detected signal cannot be interpreted as a direct representation of the vibrational response and $\chi_{CARS}^{\left(3\right)}$ can be decomposed into a resonant $\chi_{r}^{\left(3\right)}$ and a non-resonant $\chi_{nr}^{\left(3\right)}$ part

$$\chi_{CARS}^{\left(3\right)}=\chi_{r}^{\left(3\right)}+\chi_{nr}^{\left(3\right)}.$$

The resonant contribution, $\chi_{r}^{\left(3\right)}$, can be expressed as

$$\chi_{r}^{\left(3\right)}(\Omega )=\sum_{j}\frac{A_{j}}{\delta_{j}-i\Gamma_{j}},$$

where the summation runs over all vibrational modes j, $A_{j}$ denotes the oscillator strength, and $\Gamma_{j}$ represents the linewidth associated with dephasing. The detuning parameter is defined as $\delta =\Omega_{j}-(\omega_{p}-\omega_{S})$, and the resonant response is maximized when δ=0, i.e., when the pump–Stokes frequency difference matches a molecular vibration [30].

**Figure 3:**
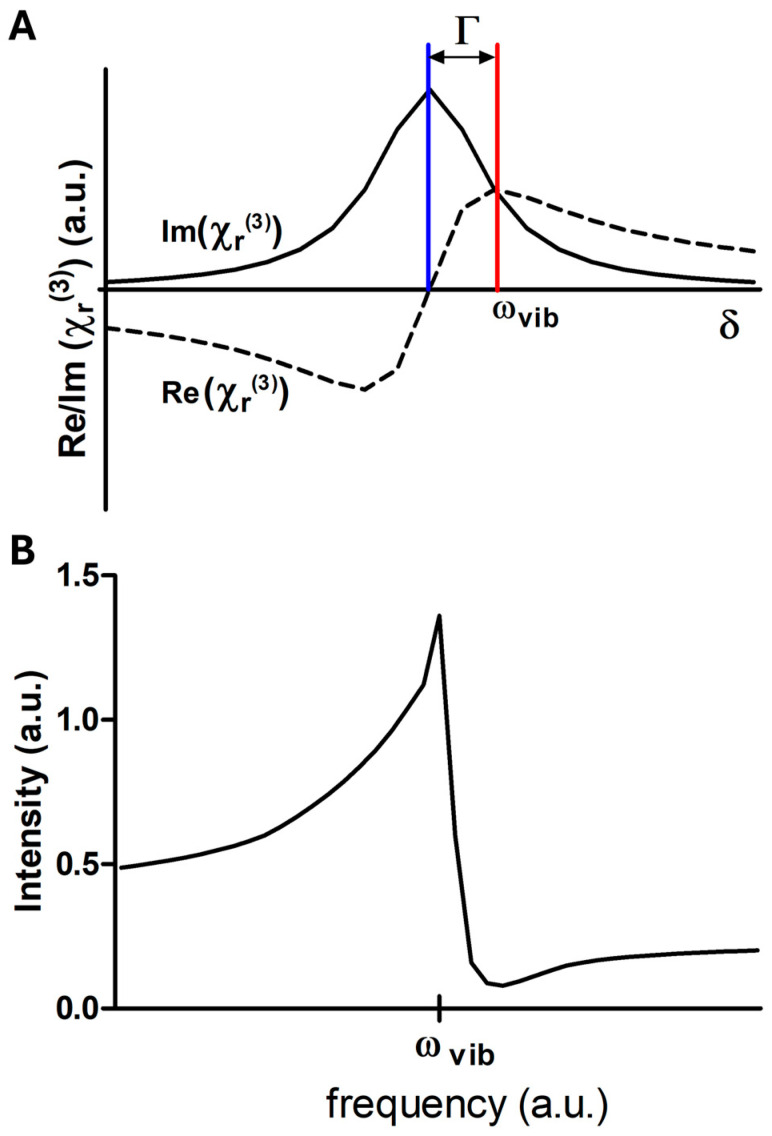
Resonant susceptibility and resulting CARS distortion: (**A**) Imaginary $Im(\chi_{r}^{\left(3\right)})$ (solid) and real $Re(\chi_{r}^{\left(3\right)})$ (dashed) components of the resonant third-order susceptibility as a function of detuning δ. The red line indicates the vibrational frequency $\omega_{vib}$, the blue line the maximum of $Im(\chi_{r}^{\left(3\right)})$, and the bracket the intrinsic linewidth Γ. (**B**) CARS intensity obtained from coherent addition of resonant and non-resonant contributions. Phase-sensitive interference between these terms produces a characteristic asymmetric profile, displaces the apparent maximum relative to $\omega_{vib}$, and generates a dip on the high-frequency side of the band. The resonant response is illustrated using a Lorentzian model. Adapted and redrawn from Ref. [30].

The resonant susceptibility is inherently complex and can be separated into real and imaginary components. The imaginary part corresponds to the Lorentzian profile that is familiar from spontaneous Raman spectroscopy, whereas the real part exhibits a dispersive lineshape, as seen in Figure 3A. While this decomposition provides a clear physical picture of the intrinsic vibrational response, the experimentally observed spectrum rarely reflects this ideal form. In practice, the resonant susceptibility interferes with the non-resonant background (NRB) present in the medium. As a result, the detected lineshapes deviate from symmetric Lorentzian profiles and instead become distorted, dispersive, and shifted in frequency, as seen in Figure 3B.

Taking these considerations into account, the detected CARS intensity can be written as

$$I_{CARS}\propto {|\chi_{r}^{\left(3\right)}+\chi_{nr}^{\left(3\right)}|}^{2}={\left(\chi_{nr}^{\left(3\right)}\right)}^{2}+2\;\chi_{nr}^{\left(3\right)}\;Re\{\chi_{r}^{\left(3\right)}\}+Re\{\chi_{r}^{\left(3\right)}{\}}^{2}+Im\{\chi_{r}^{\left(3\right)}{\}}^{2}$$

where $\chi_{r}^{\left(3\right)}$ and $\chi_{nr}^{\left(3\right)}$ denote the resonant and non-resonant components of the third-order susceptibility, respectively. $Re\left\{\cdot \right\}$ and Im{⋅} indicate the real and imaginary parts of the complex response.

This expression separates the measured signal into distinct physical contributions. The first term represents the purely non-resonant offset, whereas the second term describes interference between the resonant and non-resonant responses and is largely responsible for spectral asymmetry and apparent peak shifts. The remaining terms originate from the intrinsic resonant susceptibility and contain the vibrational information that would be observed in the absence of background [30].

**Figure 4:**
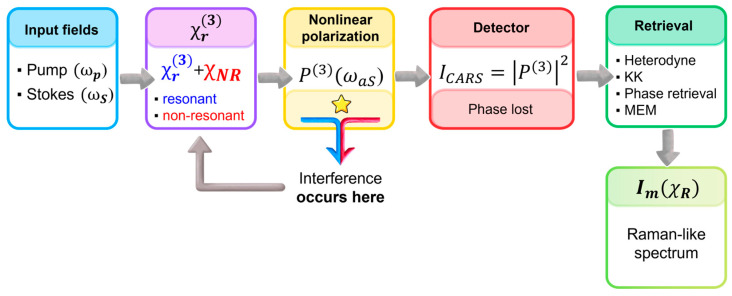
Measurement pathway from excitation to Raman-like reconstruction in CARS. Pump and Stokes fields induce a third-order nonlinear response of the medium described by $\chi^{\left(3\right)}=\chi_{R}^{\left(3\right)}+\chi_{NR}^{\left(3\right)}$. The resonant and non-resonant components combine at the level of the nonlinear polarization $P^{\left(3\right)}(\omega_{aS})$, where phase-sensitive interference occurs prior to detection. Because the measured signal is proportional to $I_{CARS}=|P^{\left(3\right)}{|}^{2}$, phase information is lost and the raw spectrum is distorted by the non-resonant background. Retrieval procedures (e.g., heterodyne detection, Kramers–Kronig analysis, phase-retrieval algorithms) reconstruct the imaginary part of the resonant susceptibility, yielding Raman-like contrast.

Several important consequences follow this interference when compared with spontaneous Raman spectroscopy. Most notably, the CARS intensity does not scale in a simple manner with the number of scatterers, because the detected signal reflects the nonlinear material response encoded in $\chi^{\left(3\right)}$. At high concentrations, quadratic contributions dominate, whereas at lower concentrations the interference term becomes relatively more pronounced and may lead to an approximately linear dependence. Furthermore, since the resonant and non-resonant components combine with a finite phase relation, the apparent maximum of a CARS band generally does not coincide with the true vibrational frequency. Instead, peaks are typically displaced toward lower energies and may exhibit a characteristic dip or suppression on the high-frequency side [23,30]. Consequently, raw CARS spectra cannot be interpreted as direct representations of the vibrational structure. As illustrated in Figure 4, interference between resonant and non-resonant contributions occurs prior to intensity detection, making dedicated retrieval procedures essential for recovering Raman-like information.

### 2.3. Practical Implementations and Strategies for Accessing the Resonant Response

The interference between resonant and non-resonant contributions, discussed in the previous section, constitutes one of the primary challenges in CARS spectroscopy. Although this mixing is intrinsic to the detection mechanism, numerous experimental concepts have been developed to enhance the visibility of the vibrational response or to recover it in a more faithful manner. Rather than altering the fundamental nonlinear interaction, these approaches exploit differences in phase behavior, polarization selection rules, temporal evolution, and excitation bandwidth to improve chemical specificity and interpretability.

Efficient generation of the anti-Stokes field requires fulfillment of the phase-matching condition,

$$k_{aS}=k_{pr}+k_{p}-k_{S},$$

where k denotes the wave vectors of the interacting fields and the subscripts aS, pr, p and S refer to the anti-Stokes, probe, pump and Stokes waves, respectively.

This condition ensures constructive buildup of the anti-Stokes radiation throughout the interaction volume [31]. In microscopic implementations, however, the requirement is substantially relaxed. Tight focusing with high numerical aperture optics and the correspondingly short propagation lengths confine the nonlinear interaction, enabling efficient signal generation even in geometries that would not fulfill strict phase matching in extended media. This relaxation has been a key factor underlying the widespread adoption of CARS in imaging.

At the same time, the non-resonant background arising from the instantaneous electronic response of the medium remains a dominant element shaping the detected spectrum. Although it carries no direct vibrational information, its coherent addition to the resonant susceptibility can mask weak modes, alter contrast, and hinder quantitative interpretation. As a result, significant methodological effort has been devoted to approaches that suppress the NRB or disentangle it from the vibrational contribution [32].

One major strategy relies on temporal discrimination. Because the electronic response is effectively instantaneous whereas vibrational coherence persists for finite dephasing times, introducing a delay between pump–Stokes excitation and probing can preferentially attenuate the background. The effectiveness of this approach is governed by the vibrational dephasing time $T_{2}$ [33], with the associated spectral linewidth given by

$$\Gamma =\frac{1}{T_{2}}.$$

Nevertheless, because the electronic and vibrational responses may partially overlap in time and because practical pulse durations are finite, temporal gating typically reduces rather than completely eliminates the non-resonant contribution.

Time-domain control therefore provides a physically intuitive handle for enhancing resonant contrast.

Another important class of strategies exploits the tensorial nature of the nonlinearnonlinear susceptibility. By tailoring excitation and detection of polarizations, selected symmetry components of the response can be emphasized while more isotropic electronic contributions are reduced. In addition to mitigating the background, polarization control enables access to molecular orientation and structural organization, thereby extending the informational content of the measurement.

Additional flexibility arises from spectral and phase engineering of the excitationexcitation of pulses. Manipulating the amplitude or phase of broadband femtosecond fields promotes constructive reinforcement of resonant pathways and selective attenuation of undesired contributions. Such coherent control approaches can substantially improve vibrational contrast and, in favorable situations, lessen the reliance on subsequent numerical retrieval.

A conceptually distinct route is provided by heterodyne or phase-sensitive detection. By interfering the anti-Stokes field with a well-defined reference, both amplitude and phase of the nonlinear susceptibility become accessible. Direct measurement of the complex response enables the separation of resonant and non-resonant components and establishes a foundation for quantitative Raman-like reconstruction [32,34].

The excitation regime itself further determines the balance between signal strength and spectral fidelity. Femtosecond excitation provides high peak intensities and broadband coverage, supporting rapid imaging and simultaneous access to multiple modes, albeit frequently at the expense of spectral resolution. Picosecond approaches, in contrast, offer narrowband selectivity and improved discrimination of individual vibrations, typically with reduced signal levels and longer acquisition times [31]. Hybrid schemes seek to combine these advantages, enabling efficient yet chemically selective operation. In biological applications, this compromise is particularly critical, as it dictates whether CARS can progress from static contrast toward resolving dynamic, mode-specific processes in living systems [32,34].

Taken together, these implementation strategies illustrate that practical CARS measurements are not rigidly determined by the underlying nonlinear interaction but can be engineered to meet specific analytical objectives. Once the resonant contribution is sufficiently enhanced or reconstructed, spectral observables can be more reliably related to material composition, structural order, and dynamics. Establishing this connection between measurement and inference forms the subject of the following section.

### 2.4. From Spectral Observables to Material Inference in CARS

Once experimental conditions are established and the resonant contribution is sufficiently enhanced or reconstructed, the central challenge becomes translating spectral observables into statements about material properties. Importantly, CARS does not measure composition, structure, or dynamics directly. Rather, these quantities are inferred from features such as band presence, intensity ratios, polarization dependence, frequency shifts, or temporal evolution. Each observable relies on underlying assumptions and is subject to potential ambiguities, which must be carefully considered to avoid overinterpretation [35,36].

Building on the measurement framework introduced in Section 2.1, this section outlines the relationship between measurable CARS signatures and material-property inference. Because detection is based on an intensity signal formed through coherent mixing of resonant and non-resonant contributions, interpretation depends not only on the chosen observable (spectral amplitude, polarization response, or time-domain behavior) but also on how the NRB and instrumental effects are treated [35,36].

**Figure 5:**
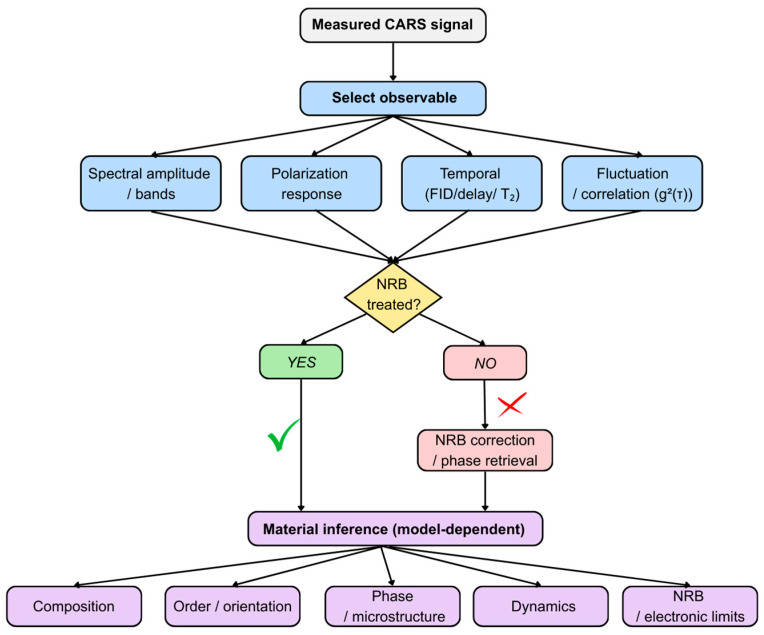
Decision workflow from CARS observables to material inference: Interpretation begins with selection of the experimentally accessible observable, including spectral features, polarization response, time-resolved behavior, or fluctuation/correlation metrics. Because the detected signal results from coherent mixing of resonant and non-resonant contributions, verification of adequate non-resonant background (NRB) treatment constitutes a mandatory step. If required, correction or phase-retrieval procedures are applied prior to further analysis. Only after this validation can the processed data be translated into material-level conclusions. The resulting inference—composition, structural order and orientation, phase or microstructure, dynamics, or recognition of electronic/NRB limitations—remains dependent on the adopted physical model.

For clarity, material inference can be organized into five principal target categories: chemical composition and identity; structural order and molecular orientation, typically accessed through polarization dependence; phase, state, and microstructure reflected in peak positions and linewidths; dynamical information derived from time-resolved or correlation-based measurements; and contributions associated with electronic or non-resonant backgrounds. For each of these targets, Table 1 summarizes the defining observable signatures, the most informative spectral regions, major confounding factors, and commonly employed acquisition or analysis strategies. Complementarily, Figure 5 presents a compact workflow from signal acquisition to interpretation, emphasizing decision points at which NRB, instrumental response, or sample heterogeneity may influence the resulting conclusions [32,37,38].

Providing an explicit mapping between measurement and inference supports reproducibility, facilitates comparison across laboratories, and clarifies the limits of what can be concluded from CARS data. With this interpretative framework in place, the technique can be more confidently applied to complex biological and material systems [32].

### 2.5. CARS in the Context of Optical Imaging Modalities

The CARS stands apart from other nonlinear vibrational imaging techniques—such as spontaneous Raman spectroscopy, SRS, or, more broadly, multi-photon imaging—primarily due to its signal generation mechanism and optical properties, as seen in Table 2. Unlike spontaneous Raman scattering, which relies on inherently weak, incoherent scattering and therefore requires long acquisition times [48], CARS is a coherent and multi-photon process: two (or more) laser beams excite a molecular vibration, and a third wave generates an anti-Stokes signal of much higher intensity [49]. Owing to its coherence, the CARS signal propagates in a well-defined direction and is more intense than in spontaneous Raman scattering, enabling rapid, high-resolution imaging [46]. At the same time, this coherence is also the origin of the characteristic nonlinear non-resonant background, which can complicate spectral interpretation—an effect does not present in either spontaneous Raman or SRS [49,50].

Compared to SRS, CARS is more susceptible to background-related artifacts and exhibits a less linear relationship between signal intensity and molecular concentration. SRS is essentially background-free, and its signal is strictly proportional to the number of vibrating molecules, making it more suitable for quantitative measurements [50,51]. CARS, on the other hand, remains advantageous in applications where high signal efficiency and straightforward spectral separation are important, thanks to the anti-Stokes shift that minimizes the influence of autofluorescence [46].

In relation to general multi-photon methods such as two-photon fluorescence or second-harmonic generation (SHG), CARS shares their benefits of deep tissue penetration, reduced photodamage, and intrinsic three-dimensional spatial confinement arising from the need for simultaneous absorption of multiple photons [52]. However, unlike two-photon fluorescence, CARS does not require any fluorescent labels; the contrast arises directly from molecular vibrations, making the method entirely label-free [46]. Moreover, in contrast to SHG or third-harmonic generation (THG), which primarily image ordered structures or interfaces, CARS provides chemically specific information, enabling differentiation of lipids, proteins, and other components based on their characteristic vibrational frequencies [36,49].

In summary, the key differences in CARS derive from the coherent nature of its signal, its high intensity and anti-Stokes shift, the presence of nonlinear background, and the fact that it is both a multi-photon and chemically specific technique. These features distinguish it from the weak but spectrally clean spontaneous Raman signal, the quantitative and background-free SRS signal, and structurally oriented but chemically non-specific multi-photon imaging methods. Raman microscopy has a major limitation: the Raman effect is extremely weak. Additionally, data acquisition times are long. Moreover, Raman microscopy requires high laser powers and long integration times of 100 ms to 1 s per pixel [36,49].

The strength of the CARS signal is proportional to the product of three incident intensities (i.e., effectively is proportional to the third power of the incident intensity), as compared to SRS, which is proportional to the product of pump and Stokes intensities (i.e., effectively is proportional to the second power of the incident intensity). This provides a better discrimination against out of focus signals as it was demonstrated in the case of three-photon absorption imaging [53].

### 2.6. Advantages and Current Limitations of CARS

CARS as a technique has many advantages. First, the test samples do not require any markers or stains. Imaging is based on the internal molecular vibrations of the sample. Moreover, as mentioned earlier, it is several orders of magnitude more sensitive than spontaneous Raman microscopy, permitting video-rate vibrational imaging at moderate excitation powers. Imagines obtained in CARS measurements characterized by high spectra resolution. By using the coherence and focusing of laser beams, CARS achieves a resolution comparable to confocal microscopy. Another advantage is the ability to obtain optical sections and 3D reconstruction of the sample structure. This is due to the fact that the CARS intensity has a quadratic dependence on the pump field intensity and a linear dependence on the Stokes field intensity, which causes the signal generated from a small volume in the central focus region [35]. Importantly, CARS is a relatively minimal invasive technique, which allows for the examination of living cells and tissue with appropriate laser power parameters. As already mentioned, but it should be emphasized, the CARS technique allows for monitoring processes in real time (e.g., lipid transport, metabolism) thanks to the high acquisition speed. Another advantage of CARS is that its signal frequency is blue-shifted from the excitation frequencies; thus, the CARS signal can be easily detected in the presence of the one-photon fluorescence background [35].

Despite its many advantages and its unique character, it must be noted that CARS technique is not without shortcomings. Because it requires the synchronization of two or more laser sources, it is a highly expensive and difficult-to-maintain system. Moreover, the intrinsically weak induced nonlinear polarizability requires sophisticated laser excitation sources with high peak power and moderate average power [35]. The microscope must operate in a stable environment to avoid wavelength or phase shifts between beams, which consequently lead to image quality impairment. When analyzing the recorded data, it should be remembered that the CARS signal contains a non-resonant component that can mask weaker band intensities and make it difficult to interpret the recorded spectra. It should also be noted that, due to its nonlinear nature and the presence of background, the signal intensity is not directly proportional to the analyte concentration. This fact indicates difficulties in quantitative analysis. Another limitation of classical CARS is the limited number of bands observed in a single measurement. However, this problem has been partially solved by the introduction of broadband CARS, which allows simultaneous examination of more than one vibration band.

## 3. Technological Advances Driving CARS

### 3.1. From a Physics-Driven Technique to a Biologically Enabling Modality

CARS microscopy has changed substantially over the past decade, mainly due to rapid technological progress. Although the basic physical principles of CARS have remained unchanged, the way this technique is implemented and applied has changed significantly. Early CARS systems were technically demanding, relied on large and complex laser configurations, had limited detection schemes, and were characterized by strong non-resonant background. These factors limited their use mainly to specialized optical laboratories and slowed their wider implementation in biological and biomedical research [54].

Recent progress has eliminated many of these limitations. The development of femtosecond and fiber laser sources has made systems more stable, flexible, and easy to use [55]. New detection geometries have made it possible for CARS imaging to work on samples that scatter a lot, like thicker tissues and in vivo models [55]. Also, using optical and computational methods to minimize NRB has significantly enhanced the quality of the spectrum and the method by which data is interpreted [56]. At the same time, the growing use of computational resources and AI has enabled the ability to analyze CARS datasets that are getting increasingly complicated with greater speed and precision.

**Figure 6:**
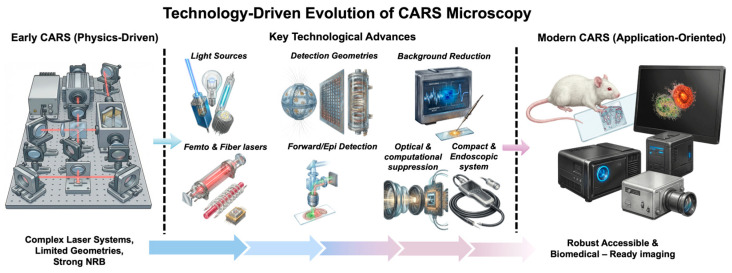
From physics-driven to application-oriented CARS: Schematic of the evolution of CARS microscopy from early benchtop, optics-heavy setups to modern, compact platforms enabled by advances in laser sources, forward/epi detection geometries, optical/computational background suppression, and miniaturization toward endoscopic and translational biomedical use.

As a result, CARS microscopy has shifted from a primarily physics-driven method toward a more practical and application-oriented imaging technique able to redefine the role of CARS in molecular biophysics and biomedicine, see Figure 6.

### 3.2. Light Sources and Laser Architectures

The performance of CARS microscopy is governed by a combination of excitation light sources, detection geometries, and strategies for mitigating NRB. Together, these technological elements determine signal strength, spectral fidelity, and the applicability of CARS to complex biological samples.

Optical parametric oscillators (OPOs) and optical parametric amplifiers (OPAs) play a crucial role in constructing tunable femtosecond CARS systems. Their spectral range is wide, and they have high peak powers [35,57,58]. Free-space ultrafast laser systems exhibit high sensitivity to environmental changes, including minor variations in temperature and humidity. Such changes may result in the beam deviating from its intended path, destabilizing the pulse, and diminishing long-term reproducibility, thereby complicating extended measurements or operations beyond strictly controlled laboratory environments [57,59]. Historically, these restrictions have limited CARS microscopy to specialized optical laboratories.

More recently, fiber-based femtosecond laser sources have emerged as a robust and viable alternative. Fiber-integrated architectures exhibit greater stability compared to free-space systems, demonstrate reduced sensitivity to environmental changes, and require less maintenance. They require less space. Turnkey fiber-based sources featuring integrated pump–Stokes synchronization have simplified daily operations and ensured long-term reliability, essential for biological imaging, longitudinal experiments, and translational research workflows [59,60].

**Figure 7:**
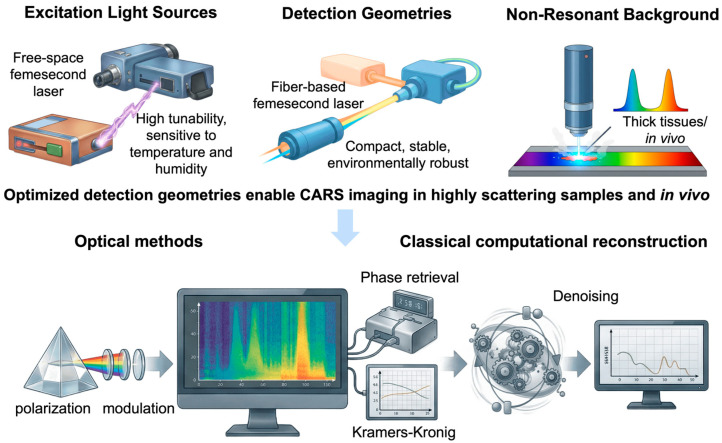
Key technological elements shaping modern CARS microscopy.

Advances in excitation sources, together with further developments in detection geometry, have been crucial to the successful application of CARS imaging to biologically relevant samples. Forward-detected CARS provides high signal efficiency in thin or weakly scattering specimens, whereas epi-detected configurations enable signal collection from highly scattering media, such as thick tissues and whole in vivo models [38,50,61]. Improvements in the detection of optics and signal collection strategies have therefore substantially expanded the range of biomedical applications accessible to CARS microscopy, see Figure 7.

### 3.3. Development of Femtosecond Lasers, Endoscopic Implementations, and Compact CARS Systems

As mentioned above, advances in femtosecond laser engineering have resulted in more compact, stable, and user-friendly ultrafast sources, reducing dependence on large free-space optical layouts and frequent realignment. In parallel, substantial effort has been made to miniaturization of CARS instrumentation.

Furthermore, one of the most groundbreaking consequences of system miniaturization was the development of endoscopic CARS. By combining femtosecond excitation with fiber optic delivery and miniaturized scanning probes, endoscopic CARS enables label-free vibrational imaging in highly scattering tissues and anatomically inaccessible areas [54,56]. Although challenges still remain in dispersion control, signal efficiency, and probe durability, recent technological advances continue to improve imaging performance and repeatability, supporting the feasibility of real-time molecular in vivo imaging [50,54,56].

### 3.4. Integration of Computational Tools and Artificial Intelligence (AI) in CARS Microscopy

Early CARS analyses concentrated on eliminating NRB and achieving phase recovery through the Kramers–Kronig (KK) relation, the Hilbert transform, and associated numerical techniques to retrieve Raman-like spectra and quantitative contrast. Physics-based methods worked, but they needed a lot of calculations and could not be used on a large scale [62,63,64]. Recent changes have made CARS more focused on high-throughput processing. Fast algorithms such as factorized KK and error correction (fKK-EC) make it possible to recover Raman data, remove noise, and correct phase and scale in real time in large hyperspectral datasets (>700,000 spectra). This is ten times faster than standard methods [58]. Hilbert transformations of the learned matrix enhance data recovery precision while preserving computational efficiency [65].

Hyperspectral CARS and SRS imaging have significantly changed how complex biological samples are analyzed. Today, advanced analytical methods such as principal component analysis (PCA), clustering, spectral unmixing, and multivariate curve resolution–alternating least squares (MCR-ALS) are routinely used to segment images and extract their underlying chemical composition. Numerous studies show that these approaches demonstrate improved performance over conventional single-feature analyses, with MCR-ALS often providing the most accurate quantitative information and enabling label-free “spectral histology” [66]. Model robustness can be enhanced through informed initialization and data-augmentation strategies [67].

**Figure 8:**
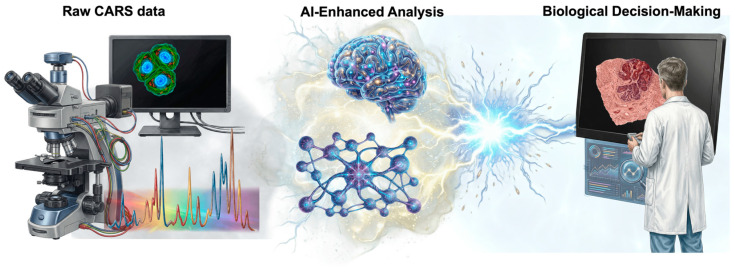
Computationally supported CARS microscopy workflow: Schematic overview of a CARS workflow supported by AI. Raw CARS data are processed to reduce noise and background and to extract chemically meaningful features, enabling automated, high-throughput interpretation such as tissue segmentation and phenotype classification.

In recent years, deep learning has become a valuable addition to classical CARS analysis. Approaches based on convolutional and recurrent networks, autoencoders, and generative models have been used to address practical challenges such as NRB removal, noise reduction, spectral separation, and data classification, in some cases enabling near real-time processing [56]. Current reviews highlight the growing interest in hybrid, physics-guided strategies, which help improve model reliability and support consistent performance across different instruments and sample types [63]. These developments enable the implementation of high-throughput, observer-independent analysis of complex biological images [39,50,52,68]. As CARS systems move toward translational and clinical applications, computational assistance will become increasingly important to support high-throughput data processing while minimizing the impact of human error, see Figure 8 [56,63,69].

## 4. CARS in Molecular Biophysics

### 4.1. CARS Microscopy as a Tool for Molecular Biophysics

Coherent excitation of molecular vibrations through synchronized pump and Stokes laser beams enables rapid, three-dimensional imaging of biological samples with high resolution and signal intensity. CARS uses vibrational Raman contrast and is not very sensitive to water, which makes it especially useful for studying biosystems that are naturally hydrated. CARS microscopy is very useful in molecular biophysics, especially when it comes to studying lipid-rich systems.

The strong CH_2_ vibrational modes facilitate the visualization of membrane architecture, lipid droplets, myelin sheaths, and lipid phase organization. CARS has been increasingly utilized to examine protein organization and aggregation, encompassing amyloid fibril formation, protein–lipid interactions, and conformational heterogeneity in dense biomolecular assemblies, in addition to its applications in lipid research. CARS offers significant insights into intracellular molecular transport and metabolism, facilitating real-time observation of molecular redistribution, storage, and turnover within living cells [36,60]. Considering the above, CARS microscopy has become a powerful tool in molecular biophysics for studying lipid metabolism, protein aggregation, and nucleic acids, all without the need for external labels. An overview of representative biological topics addressed in CARS microscopy with corresponding vibration and experimental regimes is summarized in Table 3.

It is important to note the limitations of this technique, such as the presence of NRB, and the fact that the instruments are very complicated. CARS is a very specialized and hard-to-reach technique because it needs ultra-short pulse laser sources to be perfectly synchronized.

### 4.2. Lipid Imaging by CARS

In biological samples, the stretching vibrations of C–H and C–H_2_ bonds in the high-wavenumber Raman region (about 2800–3050 cm^−1^) represent some of the strongest signals. These signals are mostly found in lipids. The high density of aliphatic chains in fatty acids, phospholipids, and neutral lipids makes CH_2_ vibrational bands natural, internal markers of lipid structures [16,70]. Using synchronized pump and Stokes laser beams to coherently excite these vibrational modes in the CARS technique makes the signal much stronger, which makes them useful for superhigh-resolution imaging.

The CARS’s high sensitivity and fast data acquisition considerably outperform conventional Raman microscopy. Due to the nonlinear and coherent nature of the generated signal, images with submicron spatial resolution can be obtained with exposure times ranging from microseconds to milliseconds, enabling the observation of dynamic processes occurring in living cells and tissues [71]. Lipid droplets (LD) are dynamic organelles responsible for the storage and transport of cholesterol and fatty acids in cells. To understand their functions and link to metabolic disorders such as obesity, type II diabetes, and atherosclerosis, it is necessary to determine the molecular composition of cells. CARS microscopy is particularly effective in visualizing lipid droplets, which play a key role in lipid storage and regulation of cellular lipid utilization. The distinct contrast generated by CH_2_ vibrations allows for precise mapping of lipid droplet distribution and enables the observation of their development, fusion, and mobilization in response to environmental changes or metabolic stimuli [72]. Multispectral techniques, such as multiplex CARS, enable the analysis of lipid saturation levels and local chain packing, which are essential in cancer and metabolic diseases [70,73]. An example illustrating the limitations of fluorescence-based techniques is their application in mammalian oocytes and preimplantation embryos, where quantitative assessment is difficult and labeling sometimes interferes with live cell imaging and normal future development. To mitigate these limitations, CARS imaging has been used in studies of mouse oocytes and preimplantation embryos [74].

Another important area of application of CARS is the investigation of biological membranes. Selective detection of CH_2_ and CH_3_ vibrational modes provides insight into lipid chain ordering, phase transitions, and the local thermodynamic state of lipid bilayers [75]. In contrast to fluorescence microscopy, CARS allows membranes to be analyzed in their native chemical composition without the introduction of exogenous probes, which is particularly important for studying delicate phase equilibria and lateral lipid organization.

**Figure 9:**
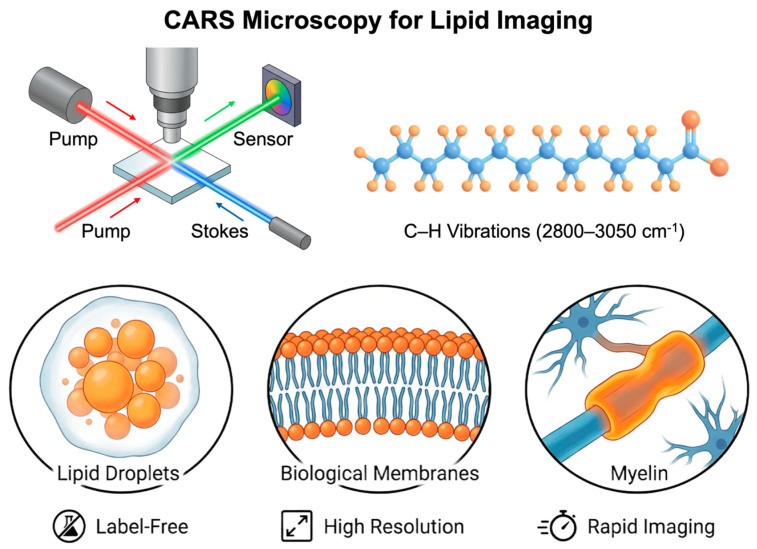
Lipid imaging by CARS in the C–H stretching region: Schematic of CARS excitation (pump and Stokes beams) and detection of strong endogenous C–H/C–H_2_ stretching vibrations (∼2800–3050 cm^−1^) that provide high-contrast, label-free chemical imaging of lipid-rich structures, including lipid droplets, biological membranes and myelin, enabling rapid acquisition with submicron spatial resolution.

An important example of CARS’ potential is the detection of myelin within the nervous system. Myelin sheaths, due to their extremely high lipid content, provide a strong CH_2_ signal, allowing their direct and label-free imaging in situ. Recent studies have demonstrated that CARS facilitates the visualization of nerve fibers, the evaluation of myelin thickness, and the examination of structural alterations linked to demyelination and neurodegeneration [76,77]. In this context, CARS offers unique capabilities that are inaccessible to conventional Raman microscopy due to the required imaging speed and signal contrast, see Figure 9.

### 4.3. Protein Imaging by CARS

Protein imaging with CARS has progressed slower than in the case of lipids, primarily due to the lower Raman cross-sections of vibrational modes associated with peptide bonds and the presence of a strong NRB. Nevertheless, early studies indicated that, with careful selection of spectral windows, amide vibrational signals can be detected—thereby opening the way to in situ, label-free imaging of protein structures [36,71]. A key milestone in this development was the identification of the amide I band (∼1650 cm^−1^) as a sensitive marker of protein secondary structure. CARS imaging in the fingerprint region was shown to enable discrimination between tissue regions exhibiting different contributions of α-helical and β-sheet conformations. The primary objective of these efforts was to translate structural information traditionally accessible through conventional Raman spectroscopy into fast, spatially resolved, label-free imaging modalities [78].

Despite these advances, protein imaging by CARS remains limited by the strong NRB in the fingerprint region. To mitigate this challenge, methodological developments such as polarization-sensitive and time-delayed CARS have been introduced, enabling enhanced amide bond contrast and selective amplification of signals arising from ordered β-sheet structures [42].

More recent work has demonstrated that coherent Raman microscopy can be effectively applied to protein imaging at the cellular level through analysis of the amide I band, extending CARS beyond its traditional lipid-dominated contrast [79]. Frequency shifts in the amide I peak associated with increased β-sheet content in disordered nucleolar proteins have enabled label-free visualization of ongoing cellular senescence. These findings underscore the potential of CARS-based techniques for probing in situ changes in the protein secondary structure and highlight new opportunities for functional, label-free imaging of dynamic biological processes. Amyloids have emerged as a particularly well-suited target for coherent Raman microscopy owing to their high degree of structural order and the predominance of β-sheet conformations, which give rise to characteristic alterations in the shape and position of the amide I band. Although many of these studies employ stimulated Raman scattering rather than CARS, their findings are highly relevant to protein imaging using coherent Raman techniques [80]. Subtle shifts in the amide I band associated with β-sheet-rich structures have been shown to enable label-free discrimination of amyloid aggregates from normal proteins in brain tissue. At the same time, these studies highlight both the promise of vibrational contrast in the fingerprint region for probing protein conformational changes and the technical challenges posed by the NRB in CARS.

Multimodal in vivo imaging techniques, integrating two-photon excitation fluorescence (TPEF) and CARS microscopy, have significantly advanced amyloid research by facilitating prolonged and repeatable visualization of amyloid-β plaques in conjunction with cerebral vasculature dynamics in animal models of Alzheimer’s disease [81]. These methods have made it possible to keep an eye on amyloid deposition and vascular morphology for long periods of time, giving us quantitative information about how plaques grow and how cerebral amyloid angiopathy gets worse as people get older. Further methodological improvements using advanced spectral analysis supported by deep learning algorithms have significantly improved the identification and classification of plaque [82]. By converting CARS spectral data into feature-rich representations and utilizing attention-based neural networks, plaque-background discrimination has significantly improved relative to traditional analytical techniques. These findings demonstrate how computational methods can enhance CARS microscopy, transforming its function from strictly longitudinal visualization to a more quantitative and automated assessment of amyloid pathology [81].

Complementary multimodal studies combining CARS microscopy with two-photon fluorescence imaging have also been applied to human Alzheimer’s disease brain tissue, revealing a pronounced spatial co-localization of β-amyloid plaques with lipid-rich structures under label-free conditions [83]. Distinct lipid morphologies within amyloid deposits, including lamellar domains and large lipid aggregates, have been identified and selectively visualized using the strong CH vibrational contrast provided by CARS. Spectral analyses further indicate that lipid signals associated with long acyl chains spatially overlap with β-sheet-rich amyloid regions, supporting the view that amyloid plaques represent heterogeneous protein–lipid assemblies rather than purely protein aggregates.

In parallel, approaches exploiting the high-wavenumber C–H stretching region (2800–3050 cm^−1^) have been developed for indirect protein imaging through the analysis of the CH_3_/CH_2_ signal ratio. As opposed to lipids, amyloid structures are characterized by higher CH_3_ signal intensity due to the tight packing of their side chains. This allows the structures to be distinguished within the cell [80].

**Figure 10:**
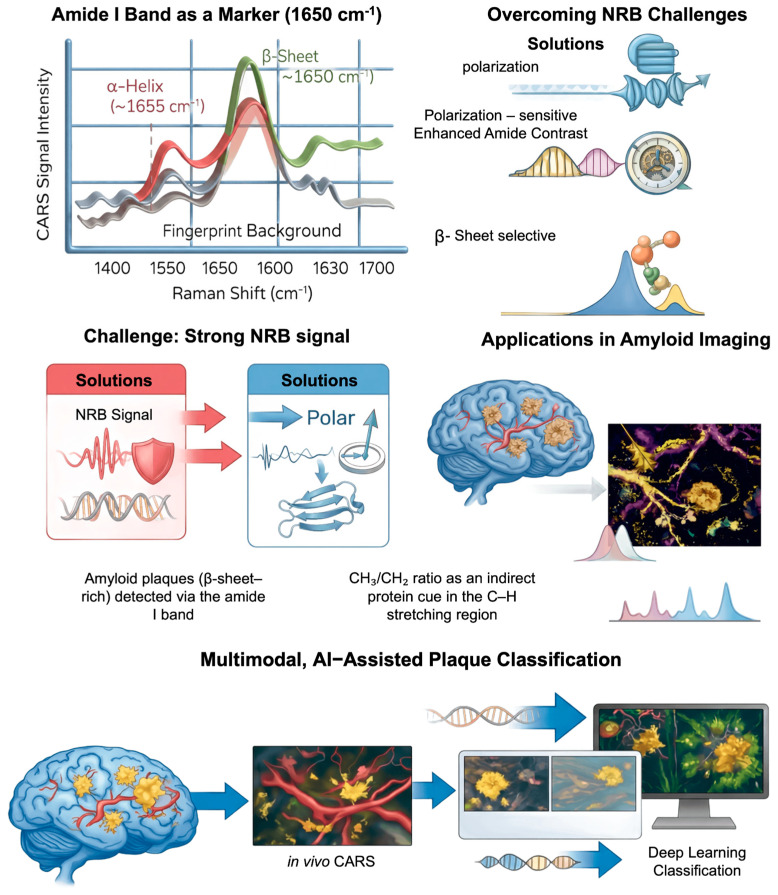
Protein imaging by CARS. Amide I-based detection of protein secondary structure (α-helix vs. β-sheet) is enabled by background-suppression strategies (polarization- and time-delayed CARS), supporting label-free visualization of β-sheet-rich amyloid plaques and multimodal in vivo imaging (CARS + TPEF) with AI-assisted plaque classification.

In general, CARS microscopy is used to image proteins for secondary structure mapping, amyloid aggregate detection, and the study of protein folding and aggregation processes in their natural environment, see Figure 10. Although its sensitivity to proteins is lower than that to lipids, improvements in background suppression, multimodal acquisition, and hyperspectral and computational analysis have made CARS an important method in structural biophysics, especially in the study of amyloid systems.

### 4.4. Chromatin and Nuclear Organization Imaging by CARS

CARS microscopy has also been employed to examine the cell nucleus and chromatin architecture, primarily through vibrational contrast in the C–H stretching region. Here, variations in the CH_3_/CH_2_ signal ratio and protein-related spectral features indicate macromolecular reorganization linked to chromatin condensation [84,85]. Multiplex and hyperspectral CARS techniques have shown that heterochromatin and condensed chromosomes can be seen without labels during the cell cycle and mitosis. This makes it possible to follow changes in the organization of the nucleus over time in both living and fixed systems [86]. Although direct, “pure” imaging of DNA in the fingerprint region is often hindered by the strong NRB inherent to CARS, the integration of spectral analysis, reconstruction, and quantitative methodologies is increasingly extending the technique toward more objective in situ mapping of chromatin condensation states [87,88].

Multiplex CARS studies performed in the high-wavenumber C–H region have shown that variations in the CH_3_/CH_2_ intensity ratio provide a sensitive readout of chromatin compaction [84]. Condensed chromatin and mitotic chromosomes exhibit distinct vibrational signatures compared to less compact nuclear regions, enabling label-free discrimination of chromatin states throughout the cell cycle and offering insights into the molecular basis of nuclear organization.

More recent work has extended CARS spectroscopy and imaging to the analysis of nucleic acids deposited on solid substrates, demonstrating that vibrational signatures of DNA in the fingerprint region can be accessed through careful spectral analysis [88]. These studies provide insight into molecular organization and nanoscale packing of nucleic acids and, although conducted on simplified model systems, highlight the potential of CARS-based approaches for probing the DNA structure. Nevertheless, realizing this potential in complex biological environments will require advanced strategies for spectral reconstruction and NRB suppression.

### 4.5. Tissue Imaging and Cancer Molecular Fingerprinting by CARS

CARS microscopy has also established itself as a powerful label-free technique for tissue-level imaging, enabling chemically specific investigations of complex biological structures, including atherosclerotic plaques, intervertebral disk components, myelin sheaths, and skin biopsy specimens [60]. In addition to structural imaging, CARS provides access to cancer-associated molecular fingerprints that reflect pathological alterations in lipid and protein organization within tissues.

Lipid metabolism in atherosclerotic tissues from both animal models and human specimens has been extensively investigated using CARS microscopy. Multimodal CARS-based imaging with picosecond laser excitation has demonstrated the ability to identify distinct types of atherosclerotic plaques solely based on intrinsic vibrational contrast, in accordance with established histopathological classification schemes [89]. The combination of CARS with sum-frequency generation (SFG) microscopy further enables quantitative assessment of lipid and collagen content across different stages of lesion development, ranging from early fatty streaks to advanced atherosclerotic plaques. Complementary studies have shown that CARS provides chemically specific, label-free visualization of cholesterol-rich domains within plaques, allowing discrimination between free cholesterol and cholesteryl ester accumulation [90]. These findings highlight the capacity of CARS to probe cholesterol metabolism and lipid organization in situ, underscoring its value for mechanistic studies of atherosclerosis progression.

In the context of neural tissues, in vivo CARS microscopy has been demonstrated as a viable approach for label-free, three-dimensional imaging of peripheral nerves. High-contrast visualization of myelinated axons has been achieved through the strong CH vibrational signature of myelin lipids. When combined with second-harmonic generation (SHG) microscopy, epi-detected CARS enables simultaneous chemical and structural imaging of nerve fibers and surrounding collagen, illustrating the suitability of CARS for minimally invasive studies of myelin-rich neural tissues [91].

Beyond cardiovascular and neural applications, multimodal vibrational and nonlinear optical imaging approaches incorporating CARS have been successfully applied to ex vivo human skin sections. These strategies enable the investigation of morpho-chemical alterations associated with basal cell carcinoma and other skin pathologies [92]. By combining CARS with second-harmonic generation, two-photon fluorescence microscopy, and spontaneous Raman spectroscopy coupled with chemometric analysis, unsupervised discrimination of carcinoma tissue from non-diseased skin can be achieved. Within such multimodal frameworks, CARS provides complementary lipid- and protein-sensitive contrasts that contribute to the tissue-level molecular fingerprinting of cancer [93].

**Figure 11:**
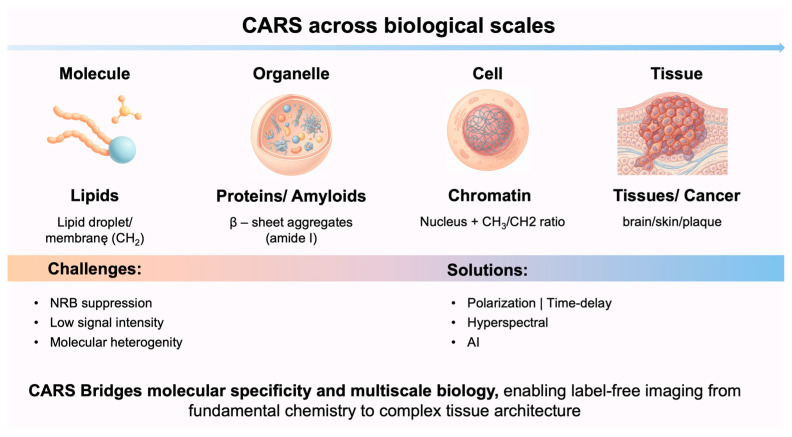
CARS bridges molecular specificity and multiscale biology. Label-free imaging platform spanning molecules (lipids, proteins), organelles, cells (chromatin), and tissues (brain, skin, cancer), overcoming technical challenges through polarization, time-delay, hyperspectral methods, and AI integration.

Overall, CARS microscopy has matured into a versatile yet continuously evolving imaging technique whose impact in biological and biomedical research is increasingly evident, see Figure 11. It enables fast, label-free visualization of lipids, proteins, chromatin, and nucleic acids, as well as chemically specific imaging of tissues and tumor models. While spontaneous Raman microscopy remains advantageous in applications where imaging speed is not critical, CARS offers distinct benefits in samples lacking second-harmonic generation contrast and in studies requiring rapid data acquisition, positioning it among the most powerful coherent Raman imaging approaches currently available.

### 4.6. CARS in Drug Discovery, Medicinal Chemistry and Bioactive Materials

CARS microscopy also acts as a chemically specific analytical method for pharmaceutical drug development, addressing critical gaps between molecular binding data, formulation behavior, and in situ biological response. In modern medical chemistry, most drug candidates with optimized affinity and selectivity fail to progress beyond the preclinical stage due to solid-state instability, formulation-dependent activity, or heterogeneous tissue effects that are not identified by conventional biophysical assays. In this context CARS and multimodal coherent Raman imaging have shown significant potential to support the decision-making process in pharmaceutical development by providing fast, label-free, and spatially resolved chemical information at various stages of the drug life cycle [94]. It has also been demonstrated that the hyperspectral CARS technique can capture drug-related chemical fingerprints in complex tissues without prior assumptions about drug localization or spectral markers. Thus, it may allow objective assessment of cellular heterogeneity and tissue-level responses in pharma research [95,96].

CARS has been shown to enable sensitive differentiation of drug polymorphism, amorphous content, and early surface crystallization in active pharmaceutical ingredients (API) and tablets at sub-micrometer resolution. This allows the detection of drug changes that may directly impact its stability, solubility and bioavailability [87]. Fossil et al. reported that in situ solubility studies using CARS indicated the link between solvent-induced phase transitions and alterations in dissolution kinetics. This effect may enable the rational optimization of pharmaceuticals’ composition as well as processing conditions rather than relying only on empirical selection [97].

Hyperspectral and quantitative CARS have also been used for mapping the distribution of drug, intracellular accumulation, and drug-induced biochemical responses in complex cellular models. Such analyses may support the early identification of heterogeneous pharmacological effects and subpopulations with poor efficacy or increased toxicity [98,99]. This capability directly complements well-established biophysical and structural techniques such as SPR, ITC, NMR, and X-ray crystallography, which provide high-resolution information on compound-target interactions, but have limited insight into composition- and context-dependent drug activity in biologically relevant environments [100].

In addition, CARS also contributes to the field of nanotechnology and biomaterial interfaces by providing label-free imaging of synthetic and polymeric materials in biological samples. In the context of pharmaceutical materials and their delivery, CARS microscopy also enables the chemical characterization of specific polymeric excipients and carrier systems used in advanced formulations. For example, label-free determination of the degree of deacetylation in chitin and chitosan provides spatial insight into polymer hydration, degradation, and drug loading properties—supporting the rational optimization of polysaccharide-based delivery systems. Of particular interest, closely related multi-color and hyperspectral CARS approaches have also enabled label-free detection and intracellular tracking of synthetic polymer particles and microplastics in cells and tissues. This is a highly sensitive and unique method that allows chemically similar materials to be clearly distinguished from endogenous lipid and protein structures [101]. Although microplastic research is often presented in an environmental and health setting, it is becoming increasingly important for pharmaceutical development, as polymer contaminants, excipient residues, and nano- or microplastic-like particles can negatively affect drug efficacy and adverse reactions at the tissue level [102,103]. Polarization-resolved CARS microscopy further extends the analytical scope of coherent Raman techniques, enabling the direct assessment of crystalline properties in solid materials. Recent work presented by Dementiev et al. has shown that polarization-sensitive CARS is able to map third-order nonlinear susceptibility associated with specific vibrational modes. It enables a clear distinction between single-crystal and structurally heterogeneous areas with spatial resolution below one micrometer [104]. Although this approach has been demonstrated on diamond needles, it may also be relevant for pharmaceutical substances, where polymorphic purity and crystallographic characteristics have a critical effect on, among other things, the stability, solubility, and bioavailability of APIs. In this regard, CARS complements conventional methods such as XRPD and DSC, especially for heterogeneous drug samples.

In the context of translational sciences, it is worth noticing that the application of CARS in organoid imaging is becoming increasingly important. Organoids are frequently used in drug research as physiologically relevant human models with cell heterogeneity and tissue-like architecture. Therefore, their complexity represents a serious challenge for standard analytical tests and imaging methods. Recently, quantitative CARS hyperspectral microscopy has been shown to enable label-free, chemically specific imaging of living organoids. It allowed the differentiation of various subpopulations of cells based on their internal biochemical composition. In liver and brain organoids, this approach revealed chemically distinct phenotypic states associated with cell cycle stage and oncogenic potential, demonstrating sensitivity to subtle biochemical differences that often are not accessible [89]. Furthermore, it should be highlighted that the application of the same method to brain-tissue transplants made it possible to distinguish between tumor and healthy tissue, as well as to differentiate between tumors derived from glioblastoma stem cells and tumors not derived from stem cells on the basis of quantitative chemical signatures. These studies underline the importance of quantitative hyperspectral CARS technology in advanced research where the identification of heterogeneous responses without the use of markers is crucial for assessing efficacy, resistance, and toxicity [96].

In summary, these advances make CARS microscopy a powerful tool for drug development, enabling earlier detection of formulation defects, heterogeneous drug responses, and interactions between materials and drugs that affect therapeutic outcomes. Thus, CARS complements classical medical chemistry processes and provides practical chemical knowledge at decision-making moments where conventional tests often fail, especially in predictions of in vivo performance.

## 5. Multimodal and Hybrid Approaches

Based on the chemical specificity and label-free nature of CARS microscopy mentioned above, recent work has been increasingly focused on pairing it with other imaging and analysis tools. Due to better optical hardware and smarter data processing, different nonlinear optical techniques can be combined within spectroscopic methods in one experimental setup, which provides the collection of structural, chemical, and functional information all at once. This kind of multimodal and hybrid strategy really pushes CARS beyond just vibrational imaging and helps the understanding of complex biological systems [36,105].

One of the most used combinations is pairing CARS with multi-photon fluorescence (MPF) microscopy. CARS provide contrast based on molecular vibrations—particularly well-suited for visualizing lipid-rich structures—while MPF allows detection of both natural tissue autofluorescence and externally introduced fluorescent markers. Regarding multi-photon excitation, MPF causes less damage to samples, better restricts the excitation area in the vertical axis, and allows deeper penetration into tissues, which works well for long-term observations of living cells. The combination of CARS and MPF thus enables direct comparison of chemical composition with biological functions in the same sample.

This mutual complementarity has been utilized in studies of host–pathogen interactions, among others. CARS serves here to visualize changes in cell membranes, lipid droplets, or nuclear structure caused by viruses, while MPF allows the precise localization of fluorescently labeled viral elements. Using the same laser source for both techniques enables synchronous data recording and direct spatial linking of biochemical changes detected by CARS with pathogen signals seen in the fluorescence channel [106].

Imaging combining CARS and MPF has also found application in studies of human skin—both in vivo and ex vivo. CARS allows for non-invasive mapping of intercellular lipid distribution in the stratum corneum, including ceramides and cholesterol, as well as visualization of water and topically applied substances. MPF adds to this by capturing natural fluorescence from keratin, NAD(P)H, melanin, and elastin, providing information about the metabolic activity and extracellular matrix arrangement [107]. Such an approach allows differentiation between healthy and diseased skin, including recognizing inflammatory and proliferative conditions, without the need for surgical biopsy.

Beyond fluorescence combinations, CARS has also been paired with other nonlinear optical techniques such as SRS, second-harmonic generation (SHG), and third-harmonic generation (THG). Platforms utilizing several methods simultaneously allow parallel observation of lipid-rich structures, collagen fibers, and protein organization, providing complementary chemical and structural contrast [108]. They are used, among other things, for assessing the myelin condition along with functional measurements in neural tissues, analyzing interactions between lipid droplets and mitochondria, determining tumor boundaries based on lipid and protein signatures, or evaluating atherosclerotic plaque stability by comparing lipid accumulation with collagen architecture.

In addition to nonlinear optical methods, attempts have been made to combine CARS with optical coherence tomography (OCT). In these hybrid setups, the chemically detailed vibrational contrast from CARS gets paired with depth-by-depth structural information from OCT. This combo allows one to correlate molecular makeup with tissue microstructure in real time, which is especially relevant for live imaging and endoscopic work with quick reads on both biochemical and structural features.

### 5.1. CARS Combined with Atomic Force Microscopy (AFM)

Beyond optical combinations, CARS microscopy has also been integrated with atomic force microscopy (AFM) to get chemically specific images at the nanoscale. Tip-enhanced CARS (TE-CARS) uses a metallic AFM tip to boost the electromagnetic field right at the sample surface, getting around the diffraction limit that normally caps how sharp conventional CARS can see things. While regular far-field CARS usually resolves features down to a few hundred nanometers, TE-CARS can do chemical imaging with resolution as fine as just a few nanometers [109,110,111,112].

In TE-CARS, an AFM tip coated with gold or silver, with an apex radius of several tens of nanometers, is illuminated by the pump and Stokes beams needed for the CARS process. Excitation of localized surface plasmons at the tip apex leads to strong amplification of the local electromagnetic field, generating an intense CARS signal from a nanometric volume directly beneath the tip. Under such conditions, background from the far field becomes negligible, giving exceptionally high spatial resolution and contrast [109].

This method has made it possible to pick up vibrational signals from individual biomolecules, including the nucleotide bases in DNA clusters, with imaging times that work for nanoscale chemical mapping. TE-CARS has also been combined with SHG and sum-frequency generation (SFG) microscopy to image cancer tissues at the nanoscale, letting researchers map protein-rich areas using the amide I band while simultaneously seeing collagen structure through SHG and SFG. By matching up mechanical properties measured with AFM and chemically specific CARS signals, these hybrid methods provide a unique window into how tissue mechanics, molecular composition, and disease state all relate to each other.

### 5.2. CARS Endoscopy

CARS microscopy has also been adapted for advanced diagnostic applications, particularly endoscopy, enabling chemically selective, real-time, and minimally invasive tissue imaging in situ without using external labels [113]. By directly exciting selected Raman resonances, CARS endoscopy provides molecular contrast unavailable to purely morphological techniques, such as optical coherence tomography alone, or to structurally specific methods, like second-harmonic generation.

**Figure 12:**
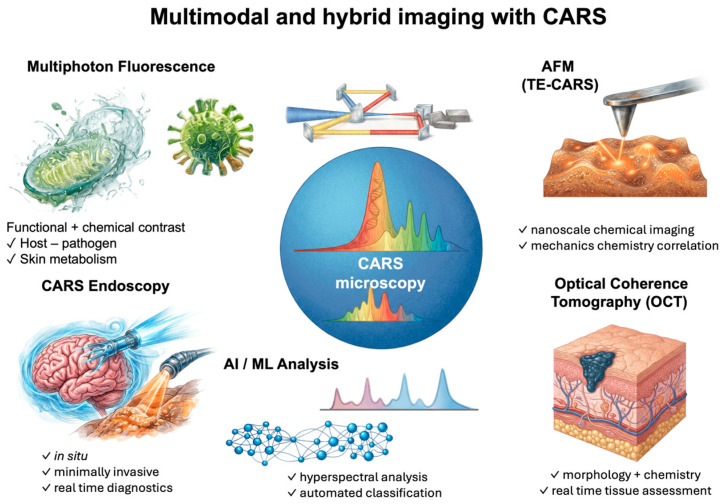
Multimodal and hybrid imaging strategies centered on CARS microscopy: CARS microscopy serves as a central platform for integration with complementary techniques, including multi-photon fluorescence microscopy, atomic force microscopy (tip-enhanced CARS), optical coherence tomography, and endoscopic imaging. Such multimodal configurations combine chemically specific vibrational contrast with functional, mechanical, and depth-resolved morphological information, extending CARS from molecular imaging toward in situ, minimally invasive, and translational biomedical applications.

Two main design strategies have been developed for CARS endoscopic systems. In solutions based on single-mode fibers, excitation beams are delivered through a single optical fiber terminated with a miniaturized focusing system. Alternatively, approaches based on multimode fibers use wavefront shaping via spatial light modulators to generate and scan a diffraction-limited focal point at the probe’s end. Thanks to the coherent nature of the CARS process, signal acquisition times are significantly shorter than in spontaneous Raman imaging, enabling rapid imaging with pixel dwell times at the millisecond level, see Figure 12 [113].

## 6. Translational Impact: From Biophysics to Medicine

**Figure 13:**
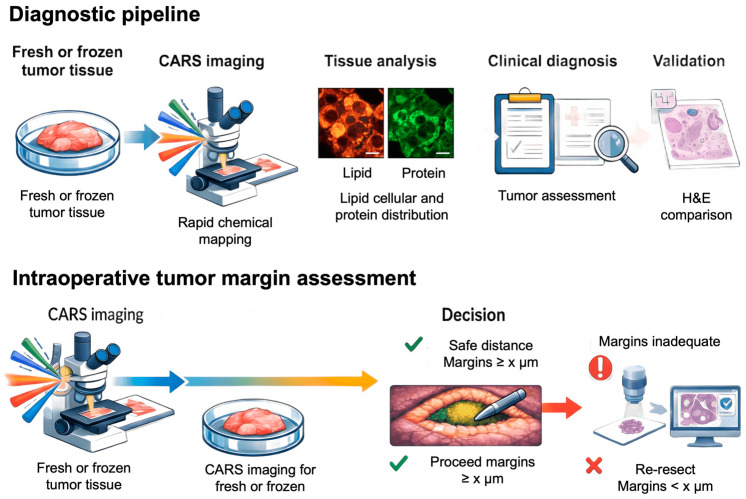
Translational diagnostic pipeline based on CARS: Rapid, label-free chemical imaging based on CH_2_/CH_3_ contrast enables assessment of fresh or frozen tissue and supports intraoperative margin evaluation, digital pathology of unstained specimens, and endoscopic or in vivo applications. CARS may be combined with TPEF and SHG, while final validation is performed using conventional H&E histology and/or Raman spectroscopy.

Classic stained histopathology still sets the diagnostic standard, but its value in the “here and now” is limited because it requires processing material for hours or days and is somewhat dependent on human interpretation [114]. CARS addresses this gap. As a nonlinear variant of Raman microspectroscopy, it provides dye-free, endogenous molecular vibration contrast and has the potential to reduce the time required to obtain chemical information to less than a minute while maintaining specificity. However, clinical implementation is not “plug-and-play”: the signal can be burdened by a strong non-resonant background arising from four-wave mixing (FWM), and fiber-based solutions that improve stability may require compromises in excitation controllability (wavelength tuning and dispersion/delay management), which are critical for point-of-care and in vivo imaging [36,114]. Photobiological safety must also be considered, because imaging endogenous nonlinear signals may require high irradiance; an increase in endogenous two-photon-excited fluorescence has been proposed as a practical indicator of developing photodamage [115]. Despite these limitations, CARS is exceptionally useful in lipid-rich tissues: the CH_2_ band (~2840 cm^−1^) enables three-dimensional, submicron visualization of myelin sheaths, even in vivo, providing a natural starting point for the translational applications discussed in this chapter [116]. Figure 13 shows the CARS translation process from biological material collection to clinically useful results, as well as the validation against H&E (hematoxylin and eosin)/Raman. This figure illustrates the three application scenarios discussed below: intraoperative margin assessment, unstained pathology/digital histology, and endoscopic and in vivo applications.

### 6.1. Intraoperative Tumor Margin Assessment

In glioma, detection of infiltrating cells during surgery is crucial for complete resection, whereas navigation based on preoperative imaging can lose precision due to brain shift during surgery. Since intraoperative diagnosis still relies on analyzing frozen sections, methods that provide microscopic insight “in situ” without time-consuming staining are actively pursued [117]. CARS has been identified as a promising tool because it enables three-dimensional, chemically selective, label-free imaging with acquisition times close to real time and can support resection margin determination and biopsy [118]. In neuro-oncology, CARS is typically tuned to CH_2_ vibrations (~2850 cm^−1^); on fresh specimens it has been shown to localize infiltrates and morphological features of glioma, such as larger nuclei, a distinct nuclear membrane, and a nucleolus, with tumor origin corroborated by GFP-TPEF in mouse models and by 5-aminolevulinic acid (5-ALA)-induced protoporphyrin IX (PpIX) fluorescence in human biopsies [117].

A CARS+TPEF workflow applied to unstained, ex vivo frozen central nervous system (CNS) sections (55 lesions) yielded images useful for typing and grading and highlighted the utility of vessel visualization for reducing bleeding risk and avoiding unrepresentative biopsies; implementation in a biopsy needle or endoscope and a penetration depth of several hundred micrometers were noted as potentially sufficient for intraoperative navigation [119]. Outside the CNS, multimodal CARS/TPEF/SHG as an adjunct to frozen section analysis in head and neck cancer showed increased TPEF/CARS contrast and 90% predictive accuracy in a four-class model [120]. In breast surgery, microcalcifications (CaP vs. CaOx) were highlighted as diagnostically relevant, and the potential of broadband CARS for rapid intraoperative screening and imaging of calcifications up to 2 mm below the surface was discussed. A limitation of margin mapping in CARS is the NRB, which hinders quantitative analysis; in a rat breast cancer model, a margin definition of ±100 μm on fresh, unstained sections was achieved within 5 min [121].

### 6.2. Label-Free Pathology and Digital Histology

In routine pathomorphology, the final diagnosis is based on evaluating H&E-stained sections, whereas rapid frozen section assessment can be more challenging and may differ from fixed and embedded preparations, motivating the development of stain-free tools for rapid ex vivo diagnosis of fresh biopsies. One approach is label-free multimodal CARS/TPEF/SHG imaging, which provides simultaneous information on architecture and biochemical composition and translates it into a format interpretable for pathologists [122]. Bocklitz et al. [122] proposed generating “pseudo-H&E” (computational H&E) using partial least squares (PLS) regression (three components) trained to map multimodal image channels to H&E RGB values; because nuclei may appear negative in multimodal data, an additional linear discriminant analysis (LDA) model was used to predict a nuclei mask that was rendered dark purple in pseudo-H&E. Background was removed automatically using k-means segmentation (k = 6), median filtering, and morphological operations, enabling rapid screening of large fields of view and identification of suspicious regions for follow-up analysis. In this workflow, regions flagged in pseudo-H&E were then examined using slower but highly specific Raman microspectroscopy (molecular “fingerprint”), achieving 100% mean sensitivity for normal vs. tumor discrimination and ~80% for differentiating normal tissue, adenoma, and carcinoma [122]. Translation of multimodal contrast into histopathological criteria was also shown in non-melanoma skin cancer (NMSC): CARS at 2850 cm^−1^ emphasized CH_2_-rich lipid signal, SHG mapped collagen, and TPEF reflected endogenous skin fluorophores; the combination enabled identification of skin layers and appendages and localization of basal cell carcinoma (BCC) and squamous cell carcinoma (SCC) nests consistent with parallel H&E, with tumor boundaries often highlighted by SHG surrounding regions of low SHG signal [123]. The authors further noted that CARS images can reveal features routinely assessed in H&E, such as peripheral palisading in BCC and keratinization features (e.g., “keratin pearls”) in well-differentiated SCC. At the cellular level, model systems support the interpretation of vibrational contrast: in CARS tuned to the CH_2_ band (~2856 cm^−1^), epi-detection provides high contrast for lipid droplets, and combining CARS with Raman enables lipid composition profiling and spectral classification using PCA followed by LDA [124].

### 6.3. Endoscopic and in Vivo Applications

The transition of CARS from tabletop platforms to in vivo and endoscopic use is driven by the need for chemically specific, label-free tissue imaging with minimal preparation under clinically relevant conditions; however, coherent Raman imaging in tissue is typically limited to the submillimeter range by absorption and scattering, making endoscopy a key route to reach deeper targets [125]. Early in vivo studies showed that CARS can deliver three-dimensional, dye-free contrast of lipid-rich structures: Huff and Cheng imaged living mouse sciatic nerves after minimally invasive exposure and combined CARS with SHG to visualize collagen; tuning to 2840 cm^−1^ (CH_2_ symmetric stretch) enabled visualization of myelin and fine features such as nodes of Ranvier and Schmidt–Lanterman incisures [91]. In scattering tissue, epi-CARS signal can originate from interfaces and from backward redirection of forward-generated components [36,91]. CARS endoscopy requires specific engineering solutions because pump and Stokes beams must be precisely overlapped in space and time, and their co-propagation in optical fibers generates parasitic FWM background [91,126]. Balu et al. showed that fiber-generated FWM can dominate unless separated and demonstrated suppression using separate fibers for excitation delivery and detection combined with dichroic optics [126]. Wang et al. proposed suppressing FWM by polarization control in polarization-maintaining fibers, reducing FWM background by ~99% without blocking return signals required for endoscopic geometry [53]. Endoscope design therefore hinges on balancing probe diameter, resolution, and signal collection efficiency in scattering tissue; Lombardini et al. presented a flexible multimodal coherent Raman endoscope (4.2 mm diameter, 71 mm rigid section) based on a double-clad Kagomé hollow-core fiber enabling low-FWM pulse delivery and efficient back-collection [125]. Miniature rigid systems are also being developed for surgical use: Zirak et al. reported a compact rigid CARS endoscope (2.2 mm diameter, 187 mm length) providing ~750 nm resolution at ~250 µm field of view (FoV) and demonstrated backscattering imaging of neurosurgically relevant tissues while emphasizing the need to control NA-dependent background in the optical path [127]. A larger endoscope described by Hirose et al. (12 mm tube diameter, 270 mm length) provided an estimated 650 µm FoV and was used to image myelinated rat sciatic nerve with low distortion and good homogeneity [128]. Collectively, these studies show that the translation of CARS to endoscopy and in vivo imaging relies on (1) detection geometries compatible with scattering tissue, (2) mitigation of fiber/system artifacts (notably FWM and dispersion), and (3) miniaturized scanning/optics that preserve resolution and FoV for real-time clinical workflows.

## 7. Challenges and Limitations

### 7.1. Non-Resonant Background (NRB)

A defining challenge of CARS is that the measured anti-Stokes intensity contains not only the vibrationally resonant third-order susceptibility, but also a non-resonant electronic contribution to $\chi^{\left(3\right)}$. Because these contributions interfere coherently, the detected CARS spectrum is not simply proportional to a spontaneous Raman spectrum: NRB can distort lineshapes, shift apparent peak positions, and introduce dispersive “dip/peak” features, reducing interpretability [78].

Importantly, NRB in CARS is not a removable additive offset but a coherent contribution that enters the observable through interference. Under intensity-only detection, the measured spectrum follows $I_{CARS}\left(\Omega \right)\propto |\chi_{NR}^{\left(3\right)}+\chi_{R}^{\left(3\right)}(\Omega )|$^2^ and naturally produces dispersive lineshape distortions and baseline reshaping. This is the physical reason why raw CARS spectra cannot be interpreted as spontaneous Raman lineshapes without phase-sensitive detection or a phase-retrieval/NRB-estimation pipeline, particularly in the fingerprint region where resonant bands are weaker [32,46].

Beyond spectral distortion, NRB directly impacts sensitivity. In practical imaging, the detector receives a large background term mixed with the resonant response; this means the shot noise (and often the effective noise floor) is set by the dominating NRB, making weak Raman bands harder to detect and quantify [78,129]. This is one reason why much early biological CARS focused on strong vibrations (e.g., CH stretching), where resonant contrast is large enough to remain visible despite NRB. Mitigation strategies (Table 4) fall into three broad categories (often combined):

(1) Optical suppression/contrast engineering. Early and still widely used approaches aim to reduce the NRB contribution at the signal formation or detection stage: (i) The review literature highlights several levers—spectral and temporal design of excitation pulses. Picosecond excitation better matches typical vibrational linewidths; femtosecond pulses can increase NRB because much of the broadband energy does not efficiently drive a single Raman resonance [60,78]. (ii) Near-infrared excitation can reduce unwanted electronic enhancement pathways and is also favored for biological specimens; Cheng’s review discusses how longer excitation wavelengths can help reduce certain background contributions while remaining off electronic resonance [78]. (iii) Geometry/detection choices (forward vs. epi). For small scatterers and heterogeneous samples, epi-detected CARS can preferentially reduce contributions that remain strongly forward-directed, which may help with background-dominated situations in certain specimen classes [78,129].

(2) Modulation/interferometric detection. Methods that encode the resonant response differently than NRB can improve contrast. A canonical example is frequency-modulation CARS (FM-CARS), which was explicitly introduced to suppress NRB and boost sensitivity to weak vibrational signatures [130]. Interferometric/heterodyne variants can also access phase information, but they add optical and calibration complexity [114].

(3) Computational phase retrieval and NRB removal. For broadband and hyperspectral CARS (including BCARS (broadband CARS)), post-processing is often essential. Standard families of algorithms recover a Raman-like response by estimating/removing NRB and retrieving phase, notably: (i) Kramers–Kronig (KK) approaches, including efficient time-domain implementations for broadband phase retrieval [39]. (ii) In modern pipelines, algorithm design and the assumed/estimated NRB shape can strongly influence spectral fidelity and cross-study comparability—one motivation for “quantitative, comparable” processing frameworks that explicitly address phase and scaling errors induced by imperfect NRB estimation [32]. (iii) More recently, deep learning approaches have been explored to automate NRB removal for BCARS; emphasizes both the need (NRB distorts peaks, reduces contrast) and the practical issue (traditional NRB removal often requires expert parameter choices and knowledge of NRB profiles) [55,131].

A key limitation of reference-based phase retrieval is that it assumes the NRB reference (often glass or water) adequately represents the sample’s effective NRB under the same optical transfer function. In practice, even modest NRB-reference mismatch can introduce slowly varying phase and amplitude errors in the recovered Raman-like spectra, reducing sample-to-sample and instrument-to-instrument comparability. Finite spectral windows and edge behavior further constrain Kramers–Kronig-type retrieval, so quantitative studies should explicitly report the spectral range, edge handling, and retrieval parameters; in many practical microspectroscopy scenarios, MEM and TDKK have been found to be functionally equivalent, implying that discrepancies often reflect data quality, windowing, and NRB estimation rather than the method label [32,38,39]

The strong NRB diminished the interpretability and quantitative accuracy of CARS signals in the fingerprint region. Thus, often it is restricted to use for protein- and nucleic acid-based phenotyping, unless it is combined with advanced phase retrieval or AI-assisted background-suppression methods.

### 7.2. Imaging Depth, Scattering, and Resolution Trade-Offs

CARS is a nonlinear, tightly focused technique with inherent 3D sectioning, yet tissue scattering and aberrations rapidly erode performance as depth increases. A practical constraint emphasized in clinical-translation discussions is that, for thick tissues, collection often relies primarily on the backward (epi) signal, because forward-propagating CARS is not accessible through bulk tissue [114]. This immediately creates a performance trade-off: forward detection is typically stronger under favorable phase-matching conditions, whereas epi detection is often weaker and depends more on sample heterogeneity, interfaces, and backscattering pathways [78,114].

Scattering also complicates quantification. Because the CARS signal scales nonlinearly with the local pump/Stokes intensities, any depth-dependent attenuation or spatial scrambling of the excitation fields changes the effective excitation conditions across the field of view, making intensity-to-concentration relationships less direct in highly scattering tissue [114]. Several interlinked “knobs” define the depth–contrast–resolution triangle: (i) Excitation wavelength: moving to longer (near-IR) wavelengths reduces scattering and can improve penetration; Cheng’s review explicitly notes that longer excitation wavelengths can reduce tissue scattering and increase penetration depth [78]. Trade-off: longer wavelengths reduce diffraction-limited spatial resolution, and they can increase dependence on specialized tunable sources. (ii) Numerical aperture (NA) and focusing conditions: high NA improves lateral/axial resolution and boosts nonlinear signal generation, but it can reduce working distance, increase sensitivity to refractive-index mismatch, and exacerbate aberrations at depth. While these optical relationships are general, they become operationally critical in CARS because signal formation is strongly intensity-dependent and coherent. (iii) Detection geometry and sample length scales: the radiation pattern depends on object size relative to the excitation wavelength; for objects much smaller than the wavelength, the forward/backward pattern becomes more symmetric, and epi-CARS can arise from small interaction lengths, interfaces, and back-reflection of forward CARS [78]. This is beneficial for certain subcellular or interfacial targets but does not eliminate scattering losses at depth.

Moreover, depth-dependent scattering and wavelength-dependent propagation can change the effective excitation and collection transfer functions across the field of view, which in turn alters the relative mixing of NRB and resonant contributions. As a result, quantitative spectral metrics may become spatially biased unless NRB referencing and normalization reflect the same optical path and scattering regime, motivating conservative interpretation and, where feasible, validation on stable standards or complementary modalities [46].

From a biomedicine perspective, these trade-offs motivate hybrid strategies, e.g., acquiring robust epi-detected contrast in vivo where forward detection is impossible, while using thin/ex vivo samples (or cleared/sectioned tissues) to recover higher fidelity hyperspectral information. Clinical-translation roadmaps explicitly discuss how adding features to overcome one barrier (e.g., higher speed, higher spectral content, interferometry) can introduce new trade-offs, underscoring the need for integrated system-level optimization rather than single-feature “upgrades” [114]. Thus, depth-dependent scattering and resolution trade-offs restrict the use of CARS in in vivo imaging and thick tissues, prompting the use of epi-detection, multimodal acquisition, and task-specific system optimization in clinical settings.

### 7.3. Costs and Standardization of Methods

Cost and system complexity remain major bottlenecks for widespread adoption, especially outside specialized photonics labs. Multiple reviews converge on the same core issue: CARS typically requires two synchronized excitation beams, with at least one being tunable to access different Raman shifts; achieving low timing jitter, stable power, and appropriate linewidth often pushes systems toward Ti:sapphire lasers plus OPOs or equivalent high-end architectures [60,114]. Li et al. explicitly summarize that the “cost and complexity” of coherent Raman microscopy are mainly driven by the laser source requirements and the need for accurate wavelength/linewidth control [60]. Tu & Boppart similarly emphasize alignment and synchronization burdens as practical barriers to broader/clinical use [114].

There is active progress toward more compact and robust sources, including fiber-based approaches intended to lower cost and improve stability. For example, a 2020 Light: Science & Applications study demonstrates a self-synchronized two-color fiber laser for coherent Raman imaging and frames such sources as enabling “wider popularization” by reducing complexity while maintaining performance [132].

**Figure 14:**
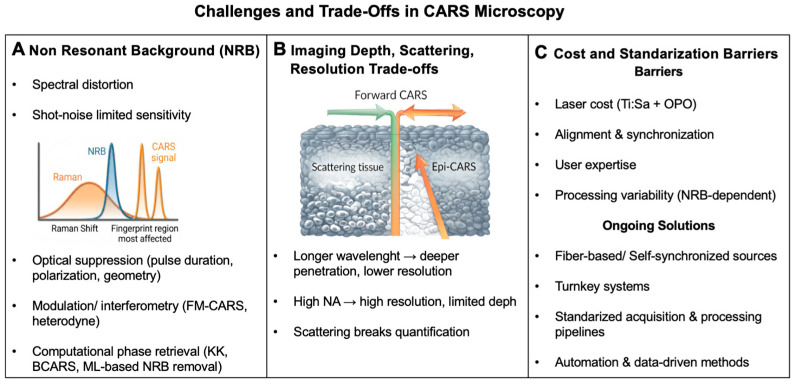
Key challenges and trade-offs in CARS microscopy: (**A**) NRB coherently interferes with the resonant response, distorting spectra and limiting sensitivity—especially in the fingerprint region; mitigation includes optical contrast engineering, modulation/heterodyne schemes, and computational phase retrieval (e.g., KK/BCARS, ML). (**B**) Imaging depth is constrained by scattering and aberrations, enforcing depth–resolution trade-offs and shifting collection from strong forward CARS to weaker but practical epi-CARS in thick tissue. (**C**) Adoption is limited by cost/complexity (dual-beam laser sources, alignment) and processing variability; progress comes from compact fiber/turnkey sources and standardized, automated acquisition/analysis pipelines.

Standardization is the second half of the translation problem: even if hardware becomes more accessible, reproducible quantitative interpretation requires consistent acquisition and processing. Two recurring pain points are: (i) NRB-dependent spectral processing and comparability: Quantitative extraction of Raman-like spectra depends on how NRB is measured/estimated and how phase retrieval is performed. Camp, Lee & Cicerone highlight that common practices (e.g., using reference NRB from glass/water) can induce amplitude/phase errors and reduce sample-to-sample and instrument-to-instrument comparability; they propose specific correction strategies to improve quantitative consistency [32]. (ii) User expertise and pipeline variability: Even modern NRB removal workflows can demand expert parameter choices; recent reviews of data-driven NRB removal stress that traditional algorithms may require specialized knowledge of NRB spectral profiles, motivating automation efforts, see Figure 14 [56]. The high cost of the system, the complexity of experiments and the variability of data processing remain major barriers to the widespread use of CARS, highlighting the need for development of standardized data acquisition protocols, robust computational processes, and ready-to-use instruments that will enable repeatable biomedical and clinical applications.

From a standardization perspective, the practical consequence is that phase retrieval alone does not automatically guarantee comparable amplitudes across sessions or instruments, because the measured intensity also embeds instrument response and the excitation spectral envelope. When quantitative cross-study comparisons are intended, additional phase/scale correction (e.g., “comparable CARS”) and transparent normalization are recommended, together with a minimal reporting checklist (NRB reference strategy, retrieval parameters, spectral window, and validation on stable standards) to make results reproducible beyond a single laboratory workflow [32,133]. To support reproducible quantitative interpretation beyond a single laboratory workflow, we summarize a practical calibration–retrieval–reporting pathway in Section 7.4.

### 7.4. Quantitative CARS in Practice: Calibration, Reporting, and NRB/Phase-Retrieval Limitations

Quantitative CARS is both enabled and constrained by the intensity-only nature of detection. As discussed in Section 2, the recorded signal arises from coherent interference between resonant and non-resonant contributions of the third-order susceptibility. Consequently, raw CARS spectra cannot be interpreted as direct representations of spontaneous Raman lineshapes, since the non-resonant background (NRB) can substantially reshape apparent peak amplitudes, positions, and baselines. Any quantitative statement must therefore explicitly define how calibration, background handling, and phase retrieval are performed [32,36,38,39].

A robust quantitative workflow begins with instrumental referencing. Frequency calibration and stability become critical whenever band positions or linewidths are interpreted, whereas power normalization and verification of linear detector response are required to prevent artefactual contrast variations arising from the nonlinear scaling of the signal. Because NRB treatment is typically the dominant source of systematic uncertainty, the choice of reference material and acquisition conditions must be documented and justified [35,134].

Recovery of Raman-like information inevitably introduces model assumptions. Methods based on time-domain Kramers–Kronig relations, maximum entropy approaches, or related strategies are sensitive to finite spectral windows, reference mismatch, and scaling conventions [38,39]. For this reason, quantitative comparisons—particularly across samples or instruments—benefit from additional phase or amplitude normalization procedures, often referred to as comparable CARS [32]. Transparent reporting of algorithmic parameters and validation criteria is essential [32,134].

Finally, uncertainties introduced at the retrieval stage propagate directly into material inference. Quantitative interpretation should therefore distinguish between relatively robust metrics, such as ratiometric trends, and strongly model-dependent quantities, including absolute concentrations or weak-feature amplitudes [32,38]. Whenever possible, independent validation against spontaneous Raman data or well-defined standards substantially strengthens confidence [32,39].

The minimal information required to ensure reproducibility and inter-study comparability is summarized in Table 5.

## 8. Future Horizons in Vibrational Biophysics

### 8.1. Molecular Movie

In ultrafast vibrational spectroscopy, one does not measure a single time-averaged spectrum, but rather a sequence of spectra recorded in successive femtosecond time windows [135]. Each spectrum encodes information about the vibrational modes active at a given moment, that is, which collective nuclear motions are excited. In practice, such observations are incomplete and noisy: not all modes are simultaneously visible, temporal resolution is finite, and parts of the signal may decay or overlap spectrally. The resulting data therefore pose an inverse inference problem, in which the time-dependent dynamics of vibrational modes must be reconstructed from fragmentary spectral observations; only when combined with computational normal-mode analysis does this reconstruction yield a physically interpretable molecular movie. Notably, in the work discussed here, machine learning techniques were employed only as an auxiliary to restrict the space of relevant vibrational degrees of freedom and to optimize tensor-network representations, rather than to directly infer experimental signals.

More broadly, time-resolved molecular experiments, including CARS, generate high-dimensional temporal datasets whose full mechanistic interpretation is often limited by experimental noise and computational cost. In this context, sequential AI models can serve as surrogate descriptions of molecular dynamics, learning temporal structure directly from data without explicitly solving the equations of motion. Recent examples—from supervised forecasting of molecular-dynamics trajectories to Koopman-based models such as VAMPnets [136] that learn conformational kinetics end-to-end—illustrate how data-driven approaches can capture underlying dynamical structure in complex molecular time series. Together, these developments highlight the suitability of AI for time-resolved vibrational data, paving the way for AI-assisted molecular movies in CARS-based spectroscopy.

### 8.2. Biobanks of Phenotyping Imaging

In classical phenotypic imaging approaches, high-throughput fluorescence microscopy is used to acquire multichannel cellular images (for example in Cell Painting assays) that encode changes in cell morphology and the spatial organization of selected subcellular structures [137]. Cellular phenotypes are subsequently represented as high-dimensional feature vectors, enabling systematic comparison of genetic and chemical perturbations and the identification of compounds that phenocopy disease-relevant gene knockouts [138]. The work discussed here demonstrates that image-based phenotypic profiling constitutes a scalable and functionally meaningful strategy for drug discovery, allowing biologically relevant cellular states to be identified without prior knowledge of molecular targets [139].

Against this backdrop, CARS microscopy emerges as a natural extension of the phenotypic imaging paradigm: rather than relying on fluorescent labels, it provides label-free chemical images that are sensitive to lipid composition and protein secondary structure, thereby enabling the definition of molecular phenotypes rooted in cellular chemistry and conformation [79]. Together, these advances suggest that, as data analysis and AI methodologies mature, CARS-based phenotypic biobanks could be constructed analogously to existing fluorescence-based imaging repositories, offering chemically resolved phenotypic references for precision medicine [140].

### 8.3. Translational Medicine

**Figure 15:**
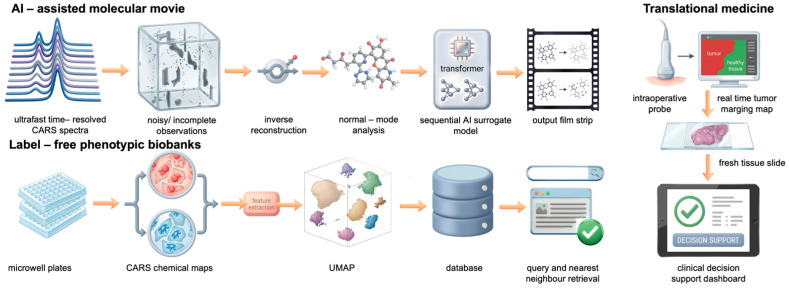
Future horizons in vibrational biophysics: Time-resolved CARS spectra can be reconstructed into AI-assisted “molecular movies” (top), while high-throughput CARS chemical maps enable label-free phenotypic biobanks for similarity search (bottom). In translational settings, coherent Raman imaging of fresh tissue supports real-time tumor-margin assessment and clinical decision support (right).

While the primary impact of time-resolved vibrational biophysics lies in defining dynamic system states, early translational implementations already illustrate its clinical relevance. In neuro-oncology, intraoperative coherent Raman-based imaging enables rapid, label-free classification of brain tumors directly during surgery [141], supporting real-time decision-making. In pancreatic surgery, label-free nonlinear optical imaging of fresh tissue provides histology-like contrast and quantitative markers of extracellular matrix organization linked to postoperative risk [142]. Together, these examples demonstrate how vibrational phenotyping can interface with clinical workflows without requiring chemical labeling or tissue processing, see Figure 15.

## 9. Summary

**Figure 16:**
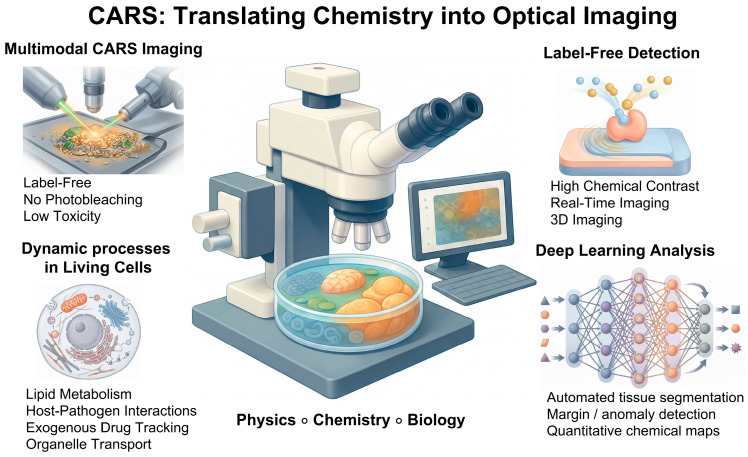
CARS microscopy converts intrinsic molecular vibrations into label-free, real-time chemical images, enabling dynamic studies in living cells and high-contrast tissue identification. By bridging physics, chemistry, and biology, CARS is evolving from a spectroscopy-based technique into a versatile platform for biophysics and biomedical imaging, extending from molecular mechanisms to clinical applications.

In summary, CARS microscopy has emerged as a powerful label-free imaging modality that provides chemically specific, real-time contrast without the need for dyes or genetic labels, see Figure 16. By exploiting the intrinsic vibrational signatures of biomolecules, CARS “translates” molecular chemistry into optical images with high sensitivity, video-rate speed and three-dimensional resolution [143]. It has been shown how advances in laser technology, detection schemes and data analysis have turned CARS from a niche nonlinear optical phenomenon into a versatile tool for molecular biophysics and biomedical research. For example, modern CARS systems can dynamically monitor cellular processes such as lipid metabolism, organelle transport, adipogenesis and host–pathogen interactions in living cells [143]. Because CARS detects molecules in their native state, it avoids photobleaching, toxicity and labeling artifacts that limit fluorescence methods, allowing truly non-perturbative studies of cell and tissue chemistry [143].

CARS’s unique contributions range from basic research on how molecules are organized to clinical imaging. In biophysics, CARS has revealed chemical heterogeneity in membranes, lipid droplets, extracellular matrices and other structures, and it can track exogenous drugs or nanoparticles in situ [143]. In biomedical applications, label-free CARS shows promise for quick pathology and live tissue diagnostics. For example, recent research showed that AI-enhanced CARS imaging of surgical specimens may identify the difference between parathyroid glands, nerves, and cancer margins without any staining [144]. Multimodal strategies—combining CARS with other nonlinear techniques such as two-photon fluorescence or second-harmonic generation—provide complementary contrast, and these co-registered datasets are ideal for high-dimensional, AI-driven analysis [7]. CARS acts as a “molecular translator,” enabling researchers to perceive the chemical state of complex samples without perturbation [143].

Looking ahead, CARS microscopy is poised to play an even bigger role in precision medicine and life-science discovery. Continued improvements in laser sources (e.g., compact fiber-based systems and supercontinuum lasers) and detection (e.g., heterodyne schemes, high-speed cameras) will make CARS instruments more robust and accessible. Integration of machine learning and computational techniques will enhance data interpretation with intelligent microscopes capable of sensing, analyzing, and adapting to revolutionize imaging [7,144]. Deep learning algorithms can be trained on hyperspectral CARS data to automatically categorize tissues or quantify molecule concentrations in vivo [144]. In parallel, miniaturized CARS probes (fiber-delivered or endoscopic) and multimodal platforms are being developed to bring real-time label-free chemistry into the clinic. Recent studies predict that label-free modalities like CARS will “win” in future intraoperative and in vivo applications, where regulatory and safety hurdles limit exogenous markers [7].

In conclusion, by bridging physics, chemistry, and biology, CARS microscopy already provides unprecedented insight into the architecture and dynamics of living matter. As technological and computational advances continue, CARS is expected to unlock new levels of chemical information in cells and tissues—from tracking neuronal activity and immune cell function to guiding cancer surgery and personalized therapy. The future of CARS lies in its role as a fully noninvasive “molecular biopsy” tool: one that can rapidly image and interpret the biochemistry of life in real time, without any labels [7,143].

## Figures and Tables

**Table 1:** Measurement targets and signatures in CARS microscopy.

Inference Target	Material Information Inferred	Observable Signature (What is Measured)	Typical Spectral Region(s)	Key Confounders	Recommended Acquisition/Analysis Strategy
Composition/chemical identity	Species; functional groups; mixture maps	Band positions; intensity ratios; hyperspectral contrast (often after retrieval)	Fingerprint; CH-stretch region	NRB *; windowing; instrument response; scattering	Multiplex/broadband; KK/MEM *; report NRB; spectral unmixing [35,39,40,41]
Structural order/orientation	Orientation distributions; order parameters	Polarization dependence; anisotropy; tensor fits	CH-stretch region; orientation-sensitive fingerprint modes	Polarization scrambling; birefringence; depolarization; NRB mixing	Polarization-resolved CARS; calibration; symmetry/tensor modeling [42,43,44,45]
Phase/state and microstructure	Packing; order–disorder; phase transitions; local heterogeneity	Peak shifts; linewidths; band-ratios	Fingerprint (subtle); CH-stretch region (robust)	Axis drift; heating; retrieval artifacts; NRB–mismatch	Raman-like retrieval before lineshape analysis; frequency validation [44,45,46]
Dynamics (time-resolved)	Dephasing; coherence lifetimes; transient responses	Temporal decays/FID *; gated contrasts; fitted constants	Mode-specific; targeted or multiplex	Chirp/timing errors; model dependence; SNR *; photothermal effects	Delay-calibrated TR-CARS *; chirp reporting; validated kinetic models [36]
Dynamics (fluctuation or correlation)	Transport; stochastic dynamics; dynamic heterogeneity	Intensity autocorrelation; g^2^(τ) *	Targeted bands; inverse contrast regimens	Drift; motion; heterogeneity; photodamage	Correlation CARS; stable excitation; explicit model assumption [47]
NRB/electronic contributions	NRB variability; electronic resonances; comparability limits	Baseline; dispersive shape; phase/scale mismatch	All regions (often strongest in fingerprint)	Reference mismatch; instrument response; overfitting	Include NRB in forward model; document retrieval; phase/scale correction [32,46]

* Abbreviations: NRB, non-resonant background; KK, Kramers–Kronig retrieval; MEM, maximum entropy method; FID, free induction decay; SNR, signal-to-noise ratio; TR-CARS, time-resolved Coherent Anti-Stokes Raman Scattering; g^2^(τ), second-order intensity autocorrelation function.

**Table 2:** Comparison of the CARS concept with related optical imaging methods.

Aspect	Spontaneous Raman	SRS	CARS	Multi-Photon Methods (2PF, SHG, THG)
Signal generation	Incoherent, spontaneous scattering	Stimulated, coherent process	Coherent, nonlinear four-wave mixing	Nonlinear optical processes
Signal strength	Very weak	Moderate	High	High
Acquisition speed	Slow (long integration times)	Fast	Fast	Fast
Chemical specificity	High	High	High	Low—moderate
Label-free contrast	Yes	Yes	Yes	Often no (2PF), structure- dependent (SHG, THG)
Background contributions	Minimal	Negligible	Non-resonant background present	Minimal
Quantitative linearity	Linear with concentration	Linear with concentration	Nonlinear dependence	Not chemically quantitative
Autofluorescence	Possible	Possible	Strongly suppressed (anti-Stokes signal)	Common in fluorescence-based methods
Depth penetration /3D confinement	Limited	Moderate	High	High
Primary information content	Chemical composition	Chemical composition (quantitative)	Chemical composition + spatial organization	Structural/morphological contrast
Typical biological applications	Static samples, fixed cells	Quantitative live-cell imaging	Dynamic, high-speed, chemically selective imaging	Structural imaging, morphology

**Table 3:** Representative biological effects investigated by CARS microscopy with corresponding vibrational contrast, acquisition regimes, and advantages over fluorescence-based imaging.

Biological Issue	Dominant Vibration	CARS Mode	Typical Timescale	Added Value vs. Fluorescence
Lipid droplet dynamics	CH_2_ stretch	fs/ps CARS	ms–s	No photobleaching
Myelin integrity	CH_2_ stretch	epi-CARS	ms	In vivo compatibility
Protein aggregation	Amide I	ps/BCARS	s–min	Secondary structure
Chromatin condensation	CH_3_/CH_2_ ratio	hyperspectral CARS	min	Label-free nuclear states
Tumor margin detection	CH_2_/CH_3_	multimodal CARS	real-time	No staining

**Table 4:** The practical comparison of non-resonant background (NRB) suppression strategies in CARS microscopy.

Strategy	Optical /Computational	Pros	Cons	Suitable for
Time-delay CARS	Optical	Strong NRB suppression	Complex optics	Fingerprint region
Polarization CARS	Optical	Simple implementation	Limited tensor selectivity	Ordered systems
KK/fKK	Computational	Raman-like spectra	Assumptions on NRB	BCARS
Deep learning	Computational	Fast, scalable	Generalization risk	Clinical workflows

KK: Kramers–Kronig phase retrieval; fKK (fKK-EC): factorized Kramers–Kronig (with error correction).

**Table 5:** Minimal reporting checklist for reproducible quantitative CARS (items listed here represent minimal reporting requirements for enabling reproducibility and cross-study comparison).

Category	What to Report	Why it Matters /Common Failure Mode
Excitation (lasers)	Excitation wavelengths, bandwidths, repetition scheme, modality.	Defines Raman-shift axis and effective resolution; without this, spectra are not comparable across studies [46].
Pulse timing/chirp	Pulse duration at the sample; chirp or spectral focusing parameters.	Alters spectral resolution and nonlinear response, directly affecting lineshapes and quantification [46].
Optics and geometry	Powers at the sample, dwell/integration time, averaging, photodamage precautions.	Photothermal effects may mimic chemical contrast and compromise reproducibility [46].
Power and acquisition	Beam powers at the sample (per beam), integration/dwell time, averaging, scan parameters; photodamage precautions.	Dose differences (heating/photodamage) can masquerade as chemistry; required for reproducibility [133].
Detector and spectrometer	Spectrometer configuration, detector type, integration settings, background correction.	Instrument response shapes baselines and apparent spectral features [133].
Raman-shift calibration	Wavelength-axis calibration method; conversion to Raman shift; stability check (e.g., repeated reference measurement).	Mandatory when peak positions or linewidths are interpreted; prevents drift from being misread as chemistry [133].
NRB reference	NRB reference material (e.g., glass/water), when/how acquired; confirmation of matched optical conditions (objective, alignment, window, settings).	NRB-reference mismatch is a dominant source of phase/scale errors and distortions in retrieved spectra [32].
Linearity/saturation check	Verification of detector linearity; criteria used to avoid saturation; stability of alignment and power.	Nonlinearity biases retrieval and undermines quantitative interpretation [133].
Pre-processing	Background subtraction, cosmic-ray removal, denoising/smoothing with parameters.	Undisclosed filtering can alter amplitudes and linewidths, preventing reproducibility [133].
Phase retrieval	Method (TDKK or MEM), implementation/software, key parameters; handling of finite spectral window/edges.	Performance depends on spectral range and SNR; parameters must be reported to assess reliability [38,39].
Phase/scale correction (comparability)	Whether post-retrieval phase detrending and scaling (“comparable CARS”) was applied; normalization strategy.	Retrieval alone does not ensure comparability; correction mitigates systematic NRB-related errors [32].
Quantification model	Define quantitative output (ratio, fitting, multivariate unmixing); component selection/regularization; validation approach.	Band ratios may appear simple, but model assumptions must be validated to support quantitative claims [40,46,134].
Replicates and uncertainty	Replicates (sample/field/day), variability metrics, QC reference (if available).	Essential for assessing repeatability and enabling cross-study interpretation [133].

## Data Availability

No new data was created or analyzed in this study. Data sharing is not applicable to this article.

## Abbreviations

The following abbreviations are used in this manuscript:

5-ALA	5-aminolevulinic acid
AFM	Atomic force microscopy
AHA	American Heart Association
AI	Artificial intelligence
BCARS	Broadband Coherent Anti-Stokes Raman Scattering
BCC	Basal cell carcinoma
CARS	Coherent Anti-Stokes Raman Scattering
CMV	Cytomegalovirus
CNS	Central nervous system
fKK-EC	Factorized Kramers–Kronig and error correction
FM-CARS	Frequency-modulation Coherent Anti-Stokes Raman Scattering
FoV	Field of view
FWM	Four-wave mixing
GFP	Green fluorescent protein
H&E	Hematoxylin and eosin
KK	Kramers–Kronig
LDA	Linear discriminant analysis
LDs	Lipid droplets
MCR-ALS	Multivariate curve resolution–alternating least squares
MMF	Multimode fibers
MPF	Multi-photon fluorescence
NA	Numerical aperture
NLO	Multiple nonlinear optical
NMSC	Non-melanoma skin cancer
NRB	Non-resonant background
OCT	Optical coherence tomography
OPAs	Optical parametric amplifiers
OPOs	Optical parametric oscillators
PCA	Principal component analysis
PLS	Partial least squares
PpIX	Protoporphyrin IX
SCC	Squamous cell carcinoma
SFG	Sum-frequency generation
SHG	Second-harmonic generation
SLM	Spatial light modulator
SMF	Single-mode fibers
SRS	Spontaneous Raman spectroscopy
TE-CARS	Tip-enhanced Coherent Anti-Stokes Raman Scattering
THG	Third-harmonic generation
TPEF	Two-photon excitation fluorescence

## References

[1] Severins I., Joo C., van Noort J. (2022). Exploring molecular biology in sequence space: The road to next-generation single-molecule biophysics. Mol. Cell.

[2] Hell S.W., Sahl S.J., Bates M., Zhuang X., Heintzmann R., Booth M.J., Bewersdorf J., Shtengel G., Hess H., Tinnefeld P. (2015). The 2015 super-resolution microscopy roadmap. J. Phys. D Appl. Phys..

[3] Schermelleh L., Ferrand A., Huser T., Eggeling C., Sauer M., Biehlmaier O., Drummen G.P.C. (2019). Super-resolution microscopy demystified. Nat. Cell Biol..

[4] Mitrea D.M., Chandra B., Ferrolino M.C., Gibbs E.B., Tolbert M., White M.R., Kriwacki R. (2018). W Methods for physical characterization of phase separated bodies and membrane-less organelles. J. Mol. Biol..

[5] Richardson J.S., Richardson D.C. (2013). Doing molecular biophysics: Finding, naming, and picturing signal within complexity. Annu. Rev. Biophys..

[6] Barsanti L., Birindelli L., Sbrana F., Lombardi G., Gualtieri P. (2023). Advanced Microscopy Techniques for Molecular Biophysics. Int. J. Mol. Sci..

[7] Shaked N.T., Boppart S.A., Wang L.V., Popp J. (2023). Label-free biomedical optical imaging. Nat. Photonics.

[8] Pham D.L., Gillette A.A., Riendeau J., Wiech K., Guzman E.C., Datta R., Skala M.C. (2024). Perspectives on label-free microscopy of heterogeneous and dynamic biological systems. J. Biomed. Opt..

[9] Folick A., Min W., Wang M.C. (2011). Label-free imaging of lipid dynamics using Coherent Anti-stokes Raman Scattering (CARS) and Stimulated Raman Scattering (SRS) microscopy. Curr. Opin. Genet. Dev..

[10] Fan H., Park J., Kao W.C., Aksamitiene E., Chaney E., Boppart M., Boppart S.A. (2025). Real-time label-free dynamic imaging of extracellular vesicles in live tissues. Proc. SPIE.

[11] Masia F., Pope I., Watson P., Langbein W., Borri P. (2018). Bessel-Beam Hyperspectral CARS Microscopy with Sparse Sampling: Enabling High-Content High-Throughput Label-Free Quantitative Chemical Imaging. Anal. Chem..

[12] Kubota R., Tanaka W., Hamachi I. (2021). Microscopic Imaging Techniques for Molecular Assemblies: Electron, Atomic Force, and Confocal Microscopies. Chem. Rev..

[13] Hill A.H., Fu D. (2019). Cellular Imaging Using Stimulated Raman Scattering Microscopy. Anal. Chem..

[14] Hickey S.M., Ung B., Bader C., Brooks R., Lazniewska J., Johnson I.R.D., Sorvina A., Logan J., Martini C., Moore C.R. (2021). Fluorescence Microscopy-An Outline of Hardware, Biological Handling, and Fluorophore Considerations. Cells.

[15] Malard L.M., Lafeta L., Cunha R.S., Nadas R., Gadelha A., Cançado L.G., Jorio A. (2021). Studying 2D materials with advanced Raman spectroscopy: CARS, SRS and TERS. Phys. Chem. Chem. Phys..

[16] Yu Y., Ramachandran P.V., Wang M.C. (2014). Shedding new light on lipid functions with CARS and SRS microscopy. Biochim. Biophys. Acta Mol. Cell Biol. Lipids.

[17] Leighton R.E., Alperstein A.M., Frontiera R.R. (2022). Label-Free Super-Resolution Imaging Techniques. Annu. Rev. Anal. Chem..

[18] Klimas A., Gallagher B.R., Wijesekara P., Fekir S., DiBernardo E.F., Cheng Z., Stolz D.B., Cambi F., Watkins S.C., StoBrody S.L. (2023). Magnify is a universal molecular anchoring strategy for expansion microscopy. Nat. Biotechnol..

[19] Liu Z., Pouli D., Alonzo C.A., Varone A., Karaliota S., Quinn K.P., Münger K., Karalis K.P., Georgakodi I. (2018). Mapping metabolic changes by noninvasive, multiparametric, high-resolution imaging using endogenous contrast. Sci. Adv..

[20] Rowe S.P., Pomper M.G. (2022). Molecular imaging in oncology: Current impact and future directions. CA Cancer J. Clin..

[21] Chakraborty I., Mazumder N., Gogoi A., Chen M.C., Zhuo G.Y. (2024). Recent advances in label-free imaging techniques based on nonlinear optical microscopy to reveal the heterogeneity of the tumor microenvironment. Biophys. Rev..

[22] Fung A.A., Shi L. (2020). Mammalian cell and tissue imaging using Raman and coherent Raman microscopy. Wiley Interdiscip. Rev. Syst. Biol. Med..

[23] Day J.P.R., Domke K.F., Rago G., Kano H., Hamaguchi H., Vartainen E.M., Bonn M. (2011). Quantitative Coherent Anti-Stokes Raman Scattering (CARS) Microscopy. J. Phys. Chem. B.

[24] Terhune R.W., Maker P.D., Savage C.M. (1965). Measurements of Nonlinear Light Scattering. Phys. Rev. Lett..

[25] Begley R.F., Harvey A.B., Byer R.L. (1974). Coherent anti-Stokes Raman spectroscopy. Appl. Phys. Lett..

[26] Reintjes J., Duncan M.D., Manuccia T.J. (1982). Scanning coherent anti-Stokes Raman microscope. Opt. Lett..

[27] Zumbusch A., Holtom G.R., Xie X.S. (1999). Three-Dimensional Vibrational Imaging by Coherent Anti-Stokes Raman Scattering. Phys. Rev. Lett..

[28] Maker P.D., Terhune R.W. (1965). Study of Optical Effects Due to an Induced Polarization Third Order in the Electric Field Strength. Phys. Rev..

[29] Cheng J.X., Volkmer A., Book L.D., Xie X.S. (2001). An Epi-Detected Coherent Anti-Stokes Raman Scattering (E-CARS) Microscope with High Spectral Resolution and High Sensitivity. J. Phys. Chem. B.

[30] Burkacky O., Zumbusch A. (2007). Coherent Anti-Stokes Raman Scattering (CARS) Microscopy. Biomedical Vibrational Spectroscopy.

[31] Roy S., Gord J.R., Patnaik A.K. (2010). Recent advances in coherent anti-Stokes Raman scattering spectroscopy: Fundamental developments and applications in reacting flows. Prog. Energy Combust. Sci..

[32] Camp C.H., Lee Y.J., Cicerone M.T. (2016). Quantitative, comparable coherent anti-Stokes Raman scattering (CARS) spectroscopy: Correcting errors in phase retrieval. J. Raman Spectrosc..

[33] Karavitis M., Zadoyan R., Apkarian V.A. (2001). Time resolved coherent anti-Stokes Raman scattering of I_2_ isolated in matrix argon: Vibrational dynamics on the ground electronic state. J. Chem. Phys..

[34] Xu S., Camp C.H., Lee Y.J. (2022). Coherent anti-Stokes Raman scattering microscopy for polymers. J. Polym. Sci..

[35] Cheng J.X., Xie X.S. (2003). Coherent Anti-Stokes Raman Scattering Microscopy: Instrumentation, Theory, and Applications. J. Phys. Chem. B.

[36] Evans C.L., Xie X.S. (2008). Coherent anti-Stokes Raman scattering microscopy: Chemical imaging for biology and medicine. Annu. Rev. Anal. Chem..

[37] Volkmer A. (2005). Vibrational imaging and microspectroscopies based on coherent anti-Stokes Raman scattering microscopy. J. Phys. D Appl. Phys..

[38] Cicerone M.T., Aamer K.A., Lee Y.J., Vartiainen E. (2012). Maximum entropy and time-domain Kramers-Kronig phase retrieval approaches are functionally equivalent for CARS microspectroscopy. J. Raman Spectrosc..

[39] Lee Y.J., Liu Y., Cicerone M.T. (2009). Broadband CARS spectral phase retrieval using a time-domain Kramers–Kronig transform. Opt. Lett..

[40] Masia F., Karuna A., Borri P., Langbein W. (2015). Hyperspectral image analysis for CARS, SRS, and Raman data. J. Raman Spectrosc..

[41] Karuna A., Masia F., Borri P., Langbein W. (2016). Hyperspectral volumetric coherent anti-Stokes Raman scattering microscopy: Quantitative volume determination and NaCl as non-resonant standard. J. Raman Spectrosc..

[42] Cheng J.-X., Book L.D., Xie X.S. (2001). Polarization coherent anti-Stokes Raman scattering microscopy. Opt. Lett..

[43] Wurpel G.W.H., Rinia H.A., Müller M. (2005). Imaging orientational order and lipid density in multilamellar vesicles with multiplex CARS microscopy. J. Microsc..

[44] Bioud F.Z., Gasecka P., Ferrand P., Rigneault H., Duboisset J., Brasselet S. (2014). Structure of molecular packing probed by polarization-resolved nonlinear four-wave mixing and coherent anti-Stokes Raman-scattering microscopy. Phys. Rev. A.

[45] Cleff C., Gasecka A., Ferrand P., Rigneault H., Brasselet S., Duboisset J. (2016). Direct imaging of molecular symmetry by coherent anti-stokes Raman scattering. Nat. Commun..

[46] Rinia H.A., Bonn M., Müller M., Vartiainen E.M. (2007). Quantitative CARS spectroscopy using the maximum entropy method: The main lipid phase transition. ChemPhysChem.

[47] Cheng J.X., Potma E.O., Xie S.X. (2002). Coherent Anti-Stokes Raman Scattering Correlation Spectroscopy: Probing Dynamical Processes with Chemical Selectivity. J. Phys. Chem. A.

[48] Long D.A. (2002). The Raman Effect: A Unified Treatment of the Theory of Raman Scattering by Molecules.

[49] Cheng J.-X., Xie X.S. (2016). Coherent Raman Scattering Microscopy.

[50] Freudiger C.W., Min W., Saar B.G., Lu S., Holtom G.R., He C., Tsai J.C., Kang J.X., Xie X.S. (2008). Label-free biomedical imaging with high sensitivity by stimulated Raman scattering microscopy. Science.

[51] Zhang D., Wang P., Slipchenko M.N., Cheng J.X. (2014). Fast Vibrational Imaging of Single Cells and Tissues by Stimulated Raman Scattering Microscopy. Acc. Chem. Res..

[52] Denk W., Strickler J.H., Webb W.W. (1990). Two-Photon Laser Scanning Fluorescence Microscopy. Science.

[53] Wang Z., Gao L., Luo P., Yang Y., Hammoudi A.A., Wong K.K., Wong S.T.C. (2011). Coherent anti-Stokes Raman scattering microscopy imaging with suppression of four-wave mixing in optical fibers. Opt. Express.

[54] Palonpon A.F., Ando J., Yamakoshi H., Dodo K., Sodeoka M., Kawata S., Fujita K. (2013). Raman and SERS microscopy for molecular imaging of live cells. Nat. Protoc..

[55] Evans C.L., Potma E.O., Puoris’haag M., Côté D., Lin C.P., Xie X.S. (2005). Chemical imaging of tissue in vivo with video-rate coherent anti-Strokes Raman scattering microscopy. Proc. Natl. Acad. Sci. USA.

[56] Vernuccio F., Broggio E., Sorrentino S., Bresci A., Junjuri R., Ventura M., Vanna R., Bocklitz T., Bregonzio M., Cerullo G. (2024). Non-resonant background removal in broadband CARS microscopy using deep-learning algorithms. Sci. Rep..

[57] Zhang C., Aldana-Mendoza J.A. (2021). Coherent Raman scattering microscopy for chemical imaging of biological systems. J. Phys. Photonics.

[58] Xu D., Liang S., Xu L., Bourdakos K.N., Johnson P., Read J., Price J.H.V., Mahajan S., Richardson D.J. (2021). Widely-tunable synchronisation-free picosecond laser source for multimodal CARS, SHG, and two-photon microscopy. Biomed. Opt. Express.

[59] Gottschall T., Meyer T., Baumgartl M., Jauregui C., Schmitt M., Popp J., Limpert J. (2015). Tünnermann Fiber-based light sources for biomedical applications of coherent anti-Stokes Raman scattering microscopy. Laser Photon. Rev..

[60] Li S., Li Y., Yi R., Liu L., Qu J. (2020). Coherent Anti-Stokes Raman Scattering Microscopy and Its Applications. Front. Phys..

[61] Siddhanta S., Kuzmin A.N., Pliss A., Baev A.S., Khare S.K., Chodhury P.K., Ganguli A.K., Prasad P.N. (2023). Advances in Raman spectroscopy and imaging for biomedical research. Adv. Opt. Photonics.

[62] Wu F., Li S., Chen X., Yue S., Hong W., Wang P. (2024). High-Sensitive and Background-Free Coherent Anti-Stokes Raman Scattering Microscopy Using Delay Modulation. Laser Photon. Rev..

[63] Junjuri R., Bocklitz T. (2025). Review of Coherent Anti-Stokes Raman Scattering Nonresonant Background Removal and Phase Retrieval Approaches: From Experimental Methods to Deep Learning Algorithms. Adv. Photonics Res..

[64] Camp C.H., Bender J.S., Lee Y.J. (2020). Real-time and high-throughput Raman signal extraction and processing in CARS hyperspectral imaging. Opt. Express.

[65] Camp C.H. (2022). Raman signal extraction from CARS spectra using a learned-matrix representation of the discrete Hilbert transform. Opt. Express.

[66] Ragupathy I.C., Schweikhard V., Zumbusch A. (2021). Multivariate analysis of hyperspectral stimulated Raman scattering microscopy images. J. Raman Spectrosc..

[67] Keating M.E., Byrne H.J. (2025). Seeding multivariate algorithms for spectral analysis, a data augmentation approach to enhance analytical performance. Spectrochim. Acta A Mol. Biomol. Spectrosc..

[68] Zhao S., Chibani L., Chandler E., Liu F., Hu J., Valzania L., Kamilov U.S., de Aguiar H.B. (2025). Computational field-resolved coherent chemical imaging. Nat. Commun..

[69] Brady A.P., Allen B., Chong J., Kotter E., Kotter M., Mongan J., Oakden-Rayner L., Pinto dos Santos D., Tang A., Wald C. (2024). Developing, purchasing, implementing and monitoring AI tools in radiology: Practical considerations. A multi-society statement from the ACR, CAR, ESR, RANZCR & RSNA. Insights Imaging.

[70] Rinia H.A., Burger K.N.J., Bonn M., Müller M. (2008). Quantitative label-free imaging of lipid composition and packing of individual cellular lipid droplets using multiplex CARS microscopy. Biophys. J..

[71] Cheng J.X., Xie X.S. (2015). Vibrational spectroscopic imaging of living systems: An emerging platform for biology and medicine. Science.

[72] Jüngst C., Winterhalder M.J., Zumbusch A. (2011). Fast and long term lipid droplet tracking with CARS microscopy. J. Biophotonics.

[73] Zhang S., He Y., Yue S. (2022). Coherent Raman scattering imaging of lipid metabolism in cancer. J. Innov. Opt. Health Sci..

[74] Bradley J., Pope I., Masia F., Sanusi R., Langbein W., Swann K., Borri P. (2016). Quantitative imaging of lipids in live mouse oocytes and early embryos using CARS microscopy. Development.

[75] Müller M., Schins J.M. (2002). Imaging the Thermodynamic State of Lipid Membranes with Multiplex CARS Microscopy. J. Phys. Chem. B.

[76] Vallée R., Côté D., Bégin S., De Koninck Y., Bélanger E., Laffray S. (2009). Quantitative myelin imaging with coherent anti-Stokes Raman scattering microscopy: Alleviating the excitation polarization dependence with circularly polarized laser beams. Opt. Express.

[77] Wang H., Fu Y., Zickmund P., Shi R., Cheng J.X. (2005). Coherent anti-stokes Raman scattering imaging of axonal myelin in live spinal tissues. Biophys. J..

[78] Cheng J.X. (2007). Coherent Anti-Stokes Raman Scattering Microscopy. Appl. Spectrosc..

[79] Ishibashi S., Inoko A., Oka Y., Leproux P., Kano H. (2024). Coherent Raman microscopy visualizes ongoing cellular senescence through amide I peak shifts originating from β sheets in disordered nucleolar proteins. Sci. Rep..

[80] Ji M., Arbrl M., Zhang L., Freudiger C.F., Hou S.S., Lin D., Yang X., Bacskai B.J., Xie X.S. (2018). Label-free imaging of amyloid plaques in Alzheimer’s disease with stimulated raman scattering microscopy. Sci. Adv..

[81] Luo Z., Xu H., Samanta S., Zhang R., Luo G., Wang Y., Liu L., Weng X., He J., Liao C. (2022). Long-Term Repeatable In Vivo Monitoring of Amyloid-β Plaques and Vessels in Alzheimer’s Disease Mouse Model with Combined TPEF/CARS Microscopy. Biomedicines.

[82] Luo Z., Zhu G., Xu H., Lin D., Li J., Qu J. (2023). Combination of deep learning and 2D CARS figures for identification of amyloid-β plaques. Opt. Express.

[83] Kiskis J., Fink H., Nyberg L., Thyr J., Li J.Y., Enejder A. (2015). Plaque-associated lipids in Alzheimer’s diseased brain tissue visualized by nonlinear microscopy. Sci. Rep..

[84] Ben T.G.-D., Rajaofara Z., Couderc V., Sol V., Kano H., Leproux P., Petit J.M. (2019). Multiplex coherent anti-Stokes Raman scattering highlights state of chromatin condensation in CH region. Sci. Rep..

[85] Pliss A., Kuzmin A.N., Kachynski A.V., Prasad P.N. (2010). Nonlinear Optical Imaging and Raman Microspectrometry of the Cell Nucleus throughout the Cell Cycle. Biophys. J..

[86] Karuna A., Masia F., Wiltshire M., Errington R., Borri P., Langbein W. (2019). Label-Free Volumetric Quantitative Imaging of the Human Somatic Cell Division by Hyperspectral Coherent Anti-Stokes Raman Scattering. Anal. Chem..

[87] Cheng J.X., Jia Y.K., Zheng G., Xie X.S. (2002). Laser-Scanning Coherent Anti-Stokes Raman Scattering Microscopy and Applications to Cell Biology. Biophys. J..

[88] Dovbeshko G., Gnatyuk O., Dementjev A., Rutkauskas D., Kovalska E., Baldycheva A., Ilchenko O., Krasnenkov D., Kaplas T. (2021). Coherent anti-stokes Raman scattering spectroscopy (CARS) and imaging of DNA on graphene layers and glass covers. FlatChem.

[89] Wang H.-W., Langohr I.M., Sturek M., Cheng J.-X. (2009). Imaging and Quantitative Analysis of Atherosclerotic Lesions by CARS-Based Multimodal Nonlinear Optical Microscopy. Arterioscler. Thromb. Vasc. Biol..

[90] Lim R.S., Suhalim J.L., Miyazaki-Anzai S., Miyazaki M., Levi M., Potma E.O., Tromberg B.J. (2011). Identification of cholesterol crystals in plaques of atherosclerotic mice using hyperspectral CARS imaging. J. Lipid Res..

[91] Huff T.B., Cheng J.X. (2007). In vivo coherent anti-Stokes Raman scattering imaging of sciatic nerve tissue. J. Microsc..

[92] Vogler N., Meyer T., Akimov D., Latka I., Krafft C., Bendose N., Svanberg K., Dietzek B., Popp J. (2010). Multimodal imaging to study the morphochemistry of basal cell carcinoma. J. Biophotonics.

[93] Messerschmidt C., Calvarese M., Vafaeinezhad M., Ryabchykov O., Mühlig A., Meyer-Zedler T., Schmitt M., Guntinas-Lichius O., Bocklitz T., Popp J. (2025). Broadband coherent Raman microspectroscopy for the investigation of head and neck cancer advancing ultrafast spectral histopathology. Discov. Imaging.

[94] Schmitt P.D. (2017). Recent Advances in Nonlinear Optical Analyses of Pharmaceutical Materials in the Solid State. Mol. Pharm..

[95] Shi J., Bera K., Mukherjee P., Alex A., Chaney E.J., Spencer-Dene B., Majer J., Marjanovic M., Spillman D.R., Hood S.R. (2023). Weakly Supervised Identification and Localization of Drug Fingerprints Based on Label-Free Hyperspectral CARS Microscopy. Anal. Chem..

[96] Pope I., Masia F., Ewan K., Jimenez-Pascual A., Dale T.C., Siebzehnrubl F.A., Borri P., Langbein W. (2021). Identifying subpopulations in multicellular systems by quantitative chemical imaging using label-free hyperspectral CARS microscopy. Analyst.

[97] Fussell A., Garbacik E., Offerhaus H., Kleinebudde P., Strachan C. (2013). In situ dissolution analysis using coherent anti-Stokes Raman scattering (CARS) and hyperspectral CARS microscopy. Eur. J. Pharm. Biopharm..

[98] Renaud J.P., Chung C., Danielson U.H., Fgner U., Hennig M., Hubbard R.E., Nar H. (2016). Biophysics in drug discovery: Impact, challenges and opportunities. Nat. Rev. Drug Discov..

[99] Dunnington E.L., Wong B.S., Fu D. (2024). Innovative Approaches for Drug Discovery: Quantifying Drug Distribution and Response with Raman Imaging. Anal. Chem..

[100] Leslie S., Berard D.J., Kamanzi A., Metera K., Scott S., Shaheen C., Shayegan M., Tahvildari R., Zhang Z. (2019). Single-molecule imaging of the biophysics of molecular interactions with precision and control, in cell-like conditions, and without tethers. Curr. Opin. Biomed. Eng..

[101] Choi D.S., Lim S., Park J.S., Kim C.H., Rhee H., Cho M. (2022). Label-Free Live-Cell Imaging of Internalized Microplastics and Cytoplasmic Organelles with Multicolor CARS Microscopy. Environ. Sci. Technol..

[102] Dementjev A., Dudoiyis V., Gelzinis A., Gylienè O. (2023). The CARS microscopy application for determination of the deacetylation degree in chitin and chitosan species. J. Raman Spectrosc..

[103] Mateos-Cárdenas A., van Pelt F.N.A.M., O’Halloran J., Jansen M.A.K. (2021). Adsorption, uptake and toxicity of micro- and nanoplastics: Effects on terrestrial plants and aquatic macrophytes. Environ. Pollut..

[104] Dementjev A., Karpicz R., Xu B., Malykhin S., Svirko Y., Kuzhir P. (2024). Polarization CARS microscopy of diamond needles. Appl. Phys. Lett..

[105] Heinrich C., Bernet S., Ritsch-Marte M. (2004). Wide-field coherent anti-Stokes Raman scattering microscopy. Appl. Phys. Lett..

[106] Robinson I., Ochsenkühn M.A., Campbell C.J., Giraud G., Hossack W.J., Arlt J., Crain J. (2010). Intracellular imaging of host-pathogen interactions using combined CARS and two-photon fluorescence microscopies. J. Biophotonics.

[107] Breunig H.G., Bückle R., Kellner-Höfer M., Weinigel M., Lademann J., Sterry W., König K. (2012). Combined in vivo multiphoton and CARS imaging of healthy and disease-affected human skin. Microsc. Res. Tech..

[108] Yue S., Slipchenko M.N., Cheng J.X. (2011). Multimodal Nonlinear Optical Microscopy. Laser Photon. Rev..

[109] Downes A., Mouras R., Elfick A. (2009). A versatile CARS microscope for biological imaging. J. Raman Spectrosc..

[110] Ichimura T., Hayazawa N., Hashimoto M., Inouye Y., Kawata S. (2004). Tip-Enhanced Coherent Anti-Stokes Raman Scattering for Vibrational Nanoimaging. Phys. Rev. Lett..

[111] Kawata S., Ichimura T., Hayazawa N., Hashimoto M., Inouye Y. (2005). Tip-enhanced near-field CARS microscopy for molecular nano-imaging. Multiphoton Microscopy in the Biomedical Sciences V.

[112] Schlücker S. (2010). Surface Enhanced Raman Spectroscopy: Analytical, Biophysical and Life Science Applications.

[113] Ganikhanov F., Légaré F., Xie X.S., Evans C.L. (2006). Towards CARS Endoscopy. Opt. Express.

[114] Tu H., Boppart S.A. (2013). Coherent anti-Stokes Raman scattering microscopy: Overcoming technical barriers for clinical translation. J. Biophotonics.

[115] Galli R., Uckermann O., Andresen E.F., Geiger K.D., Koch E., Schackert G., Steiner G., Kirsch M. (2014). Intrinsic Indicator of Photodamage during Label-Free Multiphoton Microscopy of Cells and Tissues. PLoS ONE.

[116] Fu Y., Huff T.B., Wang H.W., Wang H., Cheng J.X. (2008). Ex vivo and in vivo imaging of myelin fibers in mouse brain by coherent anti-Stokes Raman scattering microscopy. Opt. Express.

[117] Galli R., Uckermann O., Temme A., Leipnitz E., Meinhardt M., Koch E., Schackert G., Steiner G., Kirsch M. (2017). Assessing the efficacy of coherent anti-Stokes Raman scattering microscopy for the detection of infiltrating glioblastoma in fresh brain samples. J. Biophotonics.

[118] Kesari S., Wong S.T.C., Xu X., Xie X.S., Evans C.L., Young G.S. (2007). Chemically-selective imaging of brain structures with CARS microscopy. Opt. Express.

[119] Romeike B.F.M., Meyer T., Reichart R., Kalff R., Petersen I., Dietzek B., Popp J. (2015). Coherent anti-Stokes Raman scattering and two photon excited fluorescence for neurosurgery. Clin. Neurol. Neurosurg..

[120] Heuke S., Chernavskaia O., Bocklitz T., Legesse F.B., Meyer T., Akimov D., Dirsch O., Ernst G., von Eggeling F., Petersen I. (2016). Multimodal nonlinear microscopy of head and neck carcinoma—Toward surgery assisting frozen section analysis. Head Neck.

[121] Chowdary P.D., Jiang Z., Chaney E.J., Benalcazar W.A., Marks D.L., Gruebele M., Boppart S.A. (2010). Molecular histopathology by spectrally reconstructed nonlinear interferometric vibrational imaging. Cancer Res..

[122] Bocklitz T.W., Salah F.S., Vogler N., Heuke S., Chernavskaia O., Schmidt C., Waldner M.J., Greten F.R., Bräuer R., Schmitt M. (2016). Pseudo-HE images derived from CARS/TPEF/SHG multimodal imaging in combination with Raman-spectroscopy as a pathological screening tool. BMC Cancer.

[123] Heuke S., Vogler N., Meyer T., Akimov D., Kluschke F., Röwert-Huber H.J., Lademann J., Dietzek B., Popp J. (2013). Detection and Discrimination of Non-Melanoma Skin Cancer by Multimodal Imaging. Healthcare.

[124] Potcoava M.C., Futia G.L., Aughenbaugh J., Schlaepfer I.R., Gibson E.A. (2014). Raman and coherent anti-Stokes Raman scattering microscopy studies of changes in lipid content and composition in hormone-treated breast and prostate cancer cells. J. Biomed. Opt..

[125] Lombardini A., Mytskaniuk V., Sivankutty S., Andresen E.R., Chen X., Wenger J., Fabert M., Joly N., Louradour F., Kudlinski A. (2018). High-resolution multimodal flexible coherent Raman endoscope. Light Sci. Appl..

[126] Chen Z., Potma E.O., Liu G., Tromberg B.J., Balu M. (2010). Fiber delivered probe for efficient CARS imaging of tissues. Opt. Express.

[127] Zirak P., Matz G., Messerschmidt B., Meyer T., Schmitt M., Popp J., Uckermann O., Galli R., Kirsch M., Winterhalder M.J. (2018). Invited Article: A rigid coherent anti-Stokes Raman scattering endoscope with high resolution and a large field of view. APL Photonics.

[128] Hirose K., Aoki T., Furukawa T., Fukushima S., Niioka H., Deguchi S., Hashimoto M. (2018). Coherent anti-Stokes Raman scattering rigid endoscope toward robot-assisted surgery. Biomed. Opt. Express.

[129] Zhang C., Zhang D., Cheng J.X. (2015). Coherent Raman Scattering Microscopy in Biology and Medicine. Annu. Rev. Biomed. Eng..

[130] Ganikhanov F., Saar B.G., Xie X.S., Evans C.L. (2006). High-sensitivity vibrational imaging with frequency modulation coherent anti-Stokes Raman scattering (FM CARS) microscopy. Opt. Lett..

[131] Vernuccio F., Vanna R., Ceconello C., Bresci A., Manetti F., Sorrentino S., Ghislanzoni S., Lambertucci F., Motiño O., Martins I. (2023). Full-Spectrum CARS Microscopy of Cells and Tissues with Ultrashort White-Light Continuum Pulses. J. Phys. Chem. B.

[132] Kong C., Pilger C., Hachmeister H., Wei X., Cheung T.H., Lai C.S.W., Lee N.P., Tsia K.K., Wong K.K.Y., Huser T. (2020). High-contrast, fast chemical imaging by coherent Raman scattering using a self-synchronized two-colour fibre laser. Light Sci. Appl..

[133] Tsikritsis D., Legge E.J., Belsey N.A. (2022). Practical considerations for quantitative and reproducible measurements with stimulated Raman scattering microscopy. Analyst.

[134] Di Napoli C., Pope I., Masia F., Watson P., Langbein W., Borri P. (2014). Hyperspectral and differential CARS microscopy for quantitative chemical imaging in human adipocytes. Biomed. Opt. Express.

[135] Schnedermann C., Alvertis A.M., Wende T., Lukman S., Feng J., Schröder F.A.Y.N., Turban D.H.P., Wu J., Hine N.D.M., Greenham N.C. (2019). A molecular movie of ultrafast singlet fission. Nat. Commun..

[136] Liang D., Song M., Niu Z., Zhang P., Rafailovich M., Deng Y. (2021). Supervised machine learning approach to molecular dynamics forecast of SARS-CoV-2 spike glycoproteins at varying temperatures. MRS Adv..

[137] Bray M.A., Singh S., Han H., Davis C.T., Borgeson B., Hartland C., Kost-Alimova M., Gustafsdottir S.M., Gibson C.C., Carpenter A.E. (2016). Cell Painting, a high-content image-based assay for morphological profiling using multiplexed fluorescent dyes. Nat. Protoc..

[138] Bray M.A., Gustafsdottir S.M., Rohban M.H., Singh S., Ljosa V., Sokolnicki K.L., Bittker J.A., Bodycombe N.E., Dancík V., Hasaka T.P. (2017). A dataset of images and morphological profiles of 30 000 small-molecule treatments using the Cell Painting assay. GigaScience.

[139] Li B., Zhang B., Zhang C., Zhou M., Huang W., Wang S., Wang S., Li M., Zhang Y., Song Q. (2025). PhenoProfiler: Advancing phenotypic learning for image-based drug discovery. Nat. Commun..

[140] Caicedo J.C., Cooper S., Heigwer F., Warchal S., Qiu P., Molnar C., Vasilevich A.S., Barry J.D., Bansal H.S., Kraus O. (2017). Data-analysis strategies for image-based cell profiling. Nat. Methods.

[141] Orringer D.A., Pandian B., Niknafs Y.S., Hollon T.C., Boyle J., Lewis S., Garrard M., Hervey-Jumper S.L., Garton H.J.L., Maher C.O. (2017). Rapid intraoperative histology of unprocessed surgical specimens via fibre-laser-based stimulated Raman scattering microscopy. Nat. Biomed. Eng..

[142] Zhang L., Zou X., Huang J., Fan J., Sun X., Zhang B., Zheng B., Guo C., Fu D., Yao L. (2021). Label-Free Histology and Evaluation of Human Pancreatic Cancer with Coherent Nonlinear Optical Microscopy. Anal. Chem..

[143] Pezacki J.P., Blake J.A., Danielson D.C., Kennedy D.C., Lyn R.K., Singaravelu R. (2011). Chemical contrast for imaging living systems: Molecular vibrations drive CARS microscopy. Nat. Chem. Biol..

[144] Li J., Liu J., Wang Y., He Y., Liu K., Raghunathan R., Shen S.S., He T., Yu X., Danforth R. (2021). Artificial intelligence-augmented, label-free molecular imaging method for tissue identification, cancer diagnosis, and cancer margin detection. Biomed. Opt. Express.

